# Mealtime behaviours, diet quality and parental stress in children with autism spectrum disorder: a cross-sectional study from Türkiye

**DOI:** 10.1136/bmjpo-2025-004226

**Published:** 2026-06-23

**Authors:** Caner Özyildirim, Merve Seyda Karaçil Ermumcu, Meryem Ece Mutlu, Ilgın Baran, Hediye Salim, Nilgun Seremet Kürklü

**Affiliations:** 1 Department of Nutrition and Dietetics, Faculty of Health Sciences, Akdeniz University, Antalya, Turkey

**Keywords:** Child Health

## Abstract

**Background:**

Feeding difficulties in children with Autism Spectrum Disorder (ASD) are prevalent, adversely affecting both the child’s nutritional status and the family’s psychosocial well-being. This study aimed to examine the interrelationships between mealtime behaviours, diet quality and parental stress in children with ASD.

**Methods:**

A cross-sectional study was conducted with 111 parents of children diagnosed with ASD (mean age: 6.8±3.5 years) in Antalya, Türkiye. Data were collected via face-to-face interviews using validated tools (Brief Autism Mealtime Behaviour Inventory (BAMBI), parental mealtime strategies, parental stress scale, Quality of Life questionnaire in autism). Diet quality was evaluated using the Healthy Eating Index-2020, derived from 3-day food consumption records. Multivariate linear regression was used to identify independent predictors of diet quality.

**Results:**

The prevalence of poor diet quality was alarmingly high (94.5%). Multivariate regression revealed that problematic mealtime behaviours (BAMBI) were a strong independent predictor of lower diet quality (B=−0.370, p<0.001), even after adjusting for sociodemographic factors. While overall proactive parental strategies positively predicted diet quality, specific accommodative practices like preparing special meals correlated negatively. Furthermore, caregivers of girls reported significantly higher stress (p=0.017) and perceived autism-related burden (p=0.003) compared with caregivers of boys.

**Conclusions:**

Severe mealtime behavioural dysregulation in ASD intrinsically compromises diet quality and elevates parental psychosocial burden. Clinical management requires a multidisciplinary, family-centred approach integrating behavioural feeding support with caregiver stress management.

**WHAT IS ALREADY KNOWN ON THIS TOPIC**

- It is well-established that feeding difficulties are highly prevalent in children with Autism Spectrum Disorder (ASD), contributing to significant parental stress and posing risks to nutritional quality.

**WHAT THIS STUDY ADDS**

- This study reveals an exceptionally high prevalence of poor diet quality (94.5%) in a Turkish ASD sample, demonstrating that problematic child mealtime behaviours and accommodative parental feeding strategies independently worsen dietary outcomes. Furthermore, clinical stratification highlights that severe mealtime dysregulation specifically amplifies autism-related caregiver burden rather than general quality of life.

**HOW THIS STUDY MIGHT AFFECT RESEARCH, PRACTICE OR POLICY**

- Clinical management of feeding challenges in ASD must shift from isolated nutritional advice to multidisciplinary interventions that empower parents to transition from reactive feeding accommodations to structured routines while integrating caregiver stress management.

## Introduction

 Autism Spectrum Disorder (ASD) is a complex neurodevelopmental condition that significantly affects how individuals perceive, interpret, and interact with the world around them.^1^ Typically diagnosed in early childhood, ASD is characterised by persistent difficulties in reciprocal social interaction, impaired verbal and non-verbal communication, and restricted, repetitive patterns of behaviour and interests.^2^ Recent epidemiological data indicate a sharp rise in its prevalence, with approximately 2.8% of children in the US diagnosed with ASD as of 2020.^3^ Correspondingly, the prevalence of ASD in Türkiye is estimated to be 0.6% according to the most recent national screenings.^4^

Beyond the core diagnostic criteria of social communication deficits and restricted, repetitive behaviours, ASD is characterised by a complex clinical heterogeneity that frequently involves various co-occurring conditions. Among these, non-core symptoms such as gastrointestinal disturbances, heightened sensory sensitivities and marked disturbances in eating behaviour are highly prevalent and significantly contribute to the overall disease burden.^5 6^ While not part of the primary diagnostic markers, these physiological and behavioural challenges often interact with core ASD symptoms, creating a multifaceted impact on both the individual’s daily functioning and the family’s quality of life. Feeding difficulties, such as food refusal, selective eating, ritualised food behaviours and an intense preference for certain foods, are significantly more common in children with ASD compared with their typically developing peers.^7 8^ While feeding behaviours such as food selectivity are frequently observed during typical childhood development, the feeding challenges in ASD are characterised by significantly greater severity, persistence and functional impairment.^8^ In clinical contexts, these severe presentations often meet the diagnostic criteria for Paediatric Feeding Disorder (PFD)^9^ or Avoidant/Restrictive Food Intake Disorder (ARFID). However, in research using behavioural inventories, these patterns are commonly operationalised as ‘food selectivity’ or ‘food refusal’ to reflect the specific constructs measured by the assessment tools. These difficulties are often accompanied by gastrointestinal complaints such as constipation, diarrhoea, reflux and bloating,^6^ which may intensify food aversions and reinforce sensory-based avoidance patterns.^10 11^ Consequently, these feeding challenges can lead to imbalances in macro- and micronutrient intake, resulting in reduced dietary quality among children with ASD.^12 13^

Importantly, the impact of feeding-related problems in ASD extends beyond the individual child, placing substantial emotional, physical and psychological demands on caregivers. Evidence suggests that the frequency of feeding difficulties is one of the strongest predictors of the impact on caregivers’ daily lives, often outweighing the influence of core autism characteristics or sensory profiles alone.^14^ Parents of children with ASD frequently report elevated stress levels, burnout and compromised quality of life, particularly during mealtimes that are laden with conflict and anxiety.^15 16^ Recent research suggests that such parental stress does not remain isolated but may influence children’s emotional regulation, eating behaviours and the overall quality of family interactions. These challenges manifest as increased levels of caregiver worry and significant disruptions to daily routines, as parents navigate the complexities of persistent food selectivity and limited dietary variety.^14 17^ Furthermore, the severity of mealtime disturbances, as measured by tools like the Brief Autism Mealtime Behaviour Inventory (BAMBI), shows a direct positive correlation with parental anxiety and depression.^18^ This dynamic points to a bidirectional relationship in which feeding difficulties and parental psychological distress mutually reinforce one another.^19^ To manage these chronic stressors, parents frequently adopt specific feeding styles, such as emotional, instrumental or tolerance-controlled feeding, reflecting the intense pressure they feel regarding their child’s nutritional health and overall well-being.^17 20^ Such multifaceted demands often lead to altered family dynamics and chronic stress, highlighting the critical need for comprehensive intervention programmes that incorporate psychosocial support and educational strategies for caregivers to improve the overall quality of life for families living with ASD.^20 21^

Despite the high prevalence of these challenges, clinical management remains inconsistent. Addressing these challenges requires a shift from isolated behavioural interventions towards multidisciplinary, individualised approaches. Current evidence underscores that sensory processing differences, present in up to 97% of individuals with ASD, are a defining feature that shapes feeding behaviours, necessitating the integration of sensory-based interventions and Sequential Oral Sensory approaches alongside traditional behavioural techniques.^22 23^ Furthermore, in cases of co-occurring intellectual disability or severe ARFID presentations, interventions must account for significant physical and psychosocial impairments, often requiring alternative feeding strategies and intensive family involvement to maintain nutritional adequacy.^24 25^ Current guidelines from the American Academy of Paediatrics^17 26^ and the National Institute for Health and Care Excellence^27^ emphasise the necessity of nutritional surveillance but highlight a lack of standardised protocols for behavioural feeding interventions. The urgency of this gap is well-documented; while early meta-analyses established that children with ASD are five times more likely to experience feeding difficulties than their peers,^8^ recent systematic reviews confirm that these difficulties persist as chronic, severe stressors that do not spontaneously resolve with age.^28^ Furthermore, recent evidence suggests that these behaviours are major predictors of family dysregulation, often exceeding the impact of core autism symptoms.^29 30^ However, few studies have examined feeding difficulties, diet quality, parental stress, and caregiver well-being within a single integrated framework in the Turkish context.

The primary objective of this study is to examine the associations between feeding behaviours in children with ASD, their dietary quality and the subsequent psychosocial burden on their caregivers. While the co-occurrence of these challenges is recognised, the present study seeks to provide a more nuanced evaluation by identifying how specific levels of mealtime behavioural disturbances relate to various domains of parental quality of life and children’s nutritional status. By investigating these relationships, this study aims to contribute to the existing literature through a detailed analysis of the interplay between child-centred behavioural symptoms and parent-centred stress factors. The findings are expected to offer a clearer understanding of the multifaceted impact of feeding difficulties, potentially identifying critical areas where parental perceived burden and children’s diet quality are most affected. Specifically, we tested the following hypotheses:

- H1: higher scores in problematic mealtime behaviours are associated with lower diet quality.
- H2: greater parental stress is positively associated with a higher perceived autism-related burden.
- H3: controlling or accommodative parental mealtime strategies (PMAS) predict variations in children’s diet quality.

## Methods

### Study design and participants

This cross-sectional study was conducted among families of children with ASD attending special education schools in Antalya, Türkiye. Data were collected between 1 September 2022 and 1 December 2022. An a priori power analysis using G*Power determined the required sample size. The calculation was based on a bivariate correlation to align with the study’s primary aim of assessing relationships between variables. A minimum of 84 participants was required to detect a medium effect size (ρ=0.3) with 80% power at a significance level of α=0.05. The study’s final sample consisted of 111 participants from two private special education and rehabilitation centres.

In Türkiye, admission to special education and rehabilitation centres is strictly regulated by the Ministry of Health. To enrol in these centres, children are legally required to hold a valid ‘Report for Children with Special Needs’ (ÇÖZGER). Therefore, all participants included in this study had a formally verified clinical diagnosis of ASD established by qualified child psychiatrists prior to their enrolment in the special education centres. While all children in the study had a formally verified diagnosis of ASD as per national regulations, specific clinical phenotyping such as standardised ASD severity scores, cognitive functioning, functional language levels or adaptive functioning scores was not available in the rehabilitation centre records during the study period. Consequently, our findings should be interpreted as applying to a clinically heterogeneous, real-world rehabilitation sample rather than a phenotypically stratified ASD cohort. This lack of phenotypic stratification is a recognised limitation that may introduce unmeasured confounding variables.

Inclusion criteria were: (1) A formal diagnosis of ASD in the child, (2) Parental consent to participate and (3) Completion of all components of the study questionnaire. The exclusion criteria were that they were over 18 years of age. The study included children aged 1 to 17 years (mean: 6.8±3.5 years). However, the sample was heavily skewed towards early and middle childhood; 86.5% of the participants were aged 10 years or younger. This distribution reflects the typical demographic of the special education and rehabilitation centres in Türkiye. In this context, intensive support is primarily offered and structured for early childhood through health-funded services, whereas social assistance and structured programmes for adolescents or adults remain relatively more limited. During the data collection period, all parents of children with ASD attending the participating centres were invited to join the study. Those who met all inclusion criteria and provided written informed consent were enrolled using a consecutive sampling method. Regarding therapy status, the participants were largely naive to specific feeding interventions; only 6.3% of families reported having received prior nutritional education or specific behavioural feeding therapy (table 1). This allows for an assessment of feeding behaviours in a naturalistic context, unconfounded by ongoing intensive feeding treatment.

**Table 1:** Assessment of health information and dietary habits of children and families

Variables	Boys x±SD	Girls x±SD	Total	P value
Age (year)	7.2±3.5	5.6±3.4	6.8±3.5	0.051
Child’s age at diagnosis (year)	3.9±2.1	3.5±1.8	3.8±2.1	0.499
Height for age	**n (%)**	**n (%)**	**n (%)**	**χ²; P value**
Short	41 (48.2)	10 (38.5)	51 (45.9)	0.913; 0.634
Normal	28 (32.9)	11 (42.3)	39 (35.1)	
Tall	16 (18.8)	5 (19.2)	21 (18.9)	
BMI for age	**n (%)**	**n (%)**	**n (%)**	**χ²; P value**
Underweight	3 (3.6)	1 (3.8)	4	0.244*
Normal	41 (48.0)	18 (69.0)	59	
Overweight	16 (19.0)	2 (7.7)	18	
Obese	25 (29)	5 (19.0)	30	
BMI-Z Score	1.31±2.39	0.64±2.63	1.15±2.45	0.253
	**n (%**)	**n (%**)	**n (%**)	**χ²; P value**
Autism diagnosis in siblings	11 (12.9)	4 (15.4)	15 (13.5)	0.102; 0.486
Mother’s birth type				
Normal	29 (34.1)	11 (42.3)	40 (36.0)	0.579; 0.489
Caesarean	56 (65.9)	15 (57.7)	71 (64.0)	
Breastfeeding status	83 (97.6)	24 (92.3)	107 (96.4)	0.233
Breastfeeding duration	**n (%**)	**n (%**)	**n (%**)	**χ²; P value**
0−6 months	27 (31.8)	8 (30.8)	35 (31.5)	1.494; 0.474
6−12 months	11 (12.9)	3 (11.5)	14 (10.8)	
>12 months	47 (55.3)	15 (57.7)	62 (55.9)	
Comorbidities	**n (%**)	**n (%**)	**n (%**)	
None	70 (82,3)	22 (84,6)	92 (82,9)	1.000*
Epilepsy/Neurological Disorders	6 (7.0)	1 (3.8)	7 (6.3)	
Other Medical Conditions†	9 (10.7)	3 (11.6)	12 (10,8)	
	**n (%**)	**n (%**)	**n (%**)	**χ²; P value**
Food supplement use	33 (38.8)	10 (38.5)	43 (38.7)	0.000; 0.983
Food allergy	10 (11.8)	1 (3.8)	11 (9.9)	0.453*
Picky eating	58 (68.2)	20 (76.9)	78 (70.3)	0.719; 0.396
Skipping main meals	24 (28.2)	9 (34.6)	33 (29.7)	0.555; 0.456
Skipping snacks	44 (51.8)	10 (38.5)	54 (48.6)	1.410; 0.235
Ability to eat out	70 (82.4)	19 (73.6)	89 (80.2)	0.246*
Currently on a diet	8 (9.4)	3 (11.5)	11 (9.9)	0.717*
Food selectivity	54 (63.5)	18 (69.2)	72 (64.9)	0.284; 0.594
Food refusal	36 (42.4)	12 (46.2)	48 (43.2)	0.117; 0.732
Chewing-swallowing problems	17 (20.0)	5 (19.2)	22 (19.8)	0.007; 0.931
Pica	14 (16.5)	6 (23.1)	20 (18.0)	0.560*
Constipation	27 (31.8)	6 (23.1)	33 (29.7)	0.719; 0.396
Diarrhoea	7 (8.2)	2 (7.7)	9 (8.1)	1.000*
Food intolerance	5 (5.9)	–	5 (4.5)	0.589*
Temper tantrums	23 (27.1)	7 (26.9)	30 (27.0)	0.000; 0.989
Hyperactivity	50 (58.8)	13 (50.0)	63 (56.8)	0.632; 0.427
Family received child nutrition education	7 (8.2)	1 (3.8)	8 (6.3)	0.347*
Family believes they have sufficient nutrition knowledge	48 (56.6)	12 (46.2)	60 (54.1)	0.559; 0.455
Believes their child is well-nourished	41 (48.2)	10 (38.5)	51 (45.9)	0.605; 0.739

* Fisher’s exact test.

† Other Medical Conditions include single cases of anaemia, developmental delay, insulin resistance, hearing impairment, cardiac murmur, and inguinal hernia. No formal diagnoses of Attention-Deficit/Hyperactivity Disorder or Generalised Anxiety Disorder were reported in the medical history.

BMI, Body Mass Index.

### Data collection instruments

Data were collected through structured, face-to-face interviews conducted with parents by trained researchers. To minimise potential interviewer bias and social desirability bias, all researchers followed a standardised interview protocol and were trained to use neutral, non-judgmental probing questions. Participants were also assured of the confidentiality of their responses. The questionnaire comprised nine sections, as outlined below:

#### General information and dietary habits

A structured questionnaire developed by the researchers was used to collect sociodemographic data, including the child’s age, parental age, educational level and household income perception. Additionally, the questionnaire assessed the child’s clinical history and specific dietary behaviours. Variables such as the presence of food allergies, pica, constipation, diarrhoea and hyperactivity were recorded as dichotomous items (Yes/No) based on parental report. Furthermore, specific dietary habits, including meal skipping and the frequency of dining out, were assessed using multiple-choice questions. Unlike the standardised psychometric scales used in this study, this section served as a descriptive checklist for characterisation purposes and did not yield a cumulative total score. The assessment of specific dietary behaviours, such as picky eating and meal skipping, was based on parental reports using standardised definitions provided during the interview. Picky eating was defined as the consistent consumption of a limited variety of foods or the rejection of familiar/unfamiliar foods, while meal skipping referred to the child regularly missing at least one of the three primary daily meals. These dichotomous questions were used to provide a clinical characterisation of the sample’s eating patterns rather than as diagnostic tools for feeding disorders.

#### Healthy Eating Index-2020 (HEI-2020)

Participants in the study were asked to record their food consumption over a 3 day period, with 1 day falling on the weekend. Participants were provided with examples illustrating how to complete the food consumption record form. Dietary data from 3-day food records were analysed using the Computer-Aided Nutrition Programme (BeBiS), which is specifically adapted for the Turkish population and includes local recipes. To ensure accuracy in mapping Turkish mixed dishes to HEI-2020 components, recipes were decomposed into their primary ingredients. Portion sizes were estimated using standardised household measures and visual aids from the Turkey Dietary Guidelines (TÜBER) to minimise reporting bias. Dietary coding was performed by trained dietitians, and HEI-2020 was used as a comparative index to evaluate the relationship between mealtime behaviours and diet quality within this specific clinical sample. The HEI-2020 consists of 13 components covering various food groups and nutrients. Nine of these components include total fruit, whole fruit, total vegetables, greens and beans, whole grains, dairy products, total protein foods, seafood and plant-based proteins and fatty acids, which are recommended in a healthy diet. The four components that should be consumed in limited quantities are refined grains, added sugars, saturated fats and sodium. Data obtained from individuals’ food consumption records were used to calculate the index, and scoring was based on the amount of nutrients per 1000 kcal consumed. The total HEI-2020 score ranges from 0 to 100. Based on the total HEI-2020 score, ≤50 is defined as ‘poor diet quality’; the range 51–80 is defined as ‘moderate diet quality requiring improvement’ and >80 is defined as ‘good diet quality’.^31^

#### Anthropometrics

Body weight measurement was performed using a sensitive scale calibrated at regular intervals with a sensitivity of ±0.1 kg, with the individual fasting, shoes removed, wearing the lightest possible clothing, standing upright and without moving. Height was measured without shoes, with feet side by side and head in the Frankfurt plane (top of the ear and lower border of the eye socket aligned and parallel to the ground), using a wall stadiometer with a sensitivity of 0.1 cm.^32^ Body Mass Index (BMI), used for assessing thinness and obesity, was calculated using the formula [Body Weight (kg)/Height (m)^2^]. Growth status was assessed using age-specific Z-scores based on WHO-2007^33^ reference values for both age groups. For children aged 5–19 years, height and BMI Z-scores were classified into categories ranging from very tall/obese (≥2 SD) to short/very thin (<−2 SD). For children aged 2–5 years, a Z-score between −2 SD and +2 SD was considered normal for both weight-for-age and height-for-age. Scores between −2 SD and −3 SD indicated moderate growth stunting or being moderately underweight, while scores below −3 SD were classified as severe. For weight, scores between +2 SD and +3 SD suggested a risk of being overweight, and scores above +3 SD indicated a higher risk of obesity.

#### Brief autism mealtime behaviour inventory (BAMBI)

The BAMBI was originally developed by Lukens and Linscheid^34^ to assess mealtime behaviours and feeding problems in children with autism. The Turkish adaptation, validity and reliability studies were conducted by Meral and Fidan.^35^ While the original scale consisted of 18 items, the Turkish version retains 14 items after four items were eliminated during the validity analysis. The scale employs a 5-point Likert-type grading system ranging from 1 (Never/Very Rarely) to 5 (In Almost All Mealtimes), where higher scores indicate more problematic mealtime behaviours. The Turkish version identifies three sub-domains: Limited Variety, Food Refusal and Features of Autism. The internal consistency of the Turkish form was found to be satisfactory, with a Cronbach’s alpha coefficient of 0.79, and split-half reliability coefficients were calculated as 0.86 and 0.83. In the current study, the internal consistency (Cronbach’s alpha) of the BAMBI total scale for our sample was calculated as 0.651.

#### Parent mealtime action scale (PMAS)

The PMAS, originally developed by Hendy *et al*,^36^ was adapted into Turkish by Aslan and Erol^37^ to determine the relationship between childhood obesity and parental mealtime behaviours. This 31-item scale comprises nine sub-dimensions: Snack Limits, Positive Persuasion, Daily Fruit and Vegetable Availability, Use of Rewards, Insistence on Eating, Snack Modelling, Special Meals, Fat Reduction and Many Food Choices. Responses are recorded on a 3-point Likert scale (1=Never, 2=Sometimes, 3=Always), with reverse scoring applied to items 2 and 31. The subscales are scored by calculating the mean of the items within each dimension, where a higher mean indicates a higher frequency of that specific parental behaviour. Reliability analysis for the Turkish version showed Cronbach’s alpha coefficients for the sub-dimensions ranging between 0.41 and 0.75. In the current study, the Cronbach’s alpha coefficient for the total PMAS in our sample was 0.617, aligning with the acceptable reliability ranges reported in the scale’s initial Turkish adaptation.

#### Parental stress scale (PSS)

To evaluate parental stress, the PSS was developed by Özmen and Özmen^38^ to measure the stress parents experience in their daily relationships with their children, specifically within the context of primary education. Following the creation of an item pool and expert review, the final scale consists of 16 items structured as a unidimensional (single-factor) instrument. Participants respond using a 4-point Likert-type scale ranging from “Never” (1) to “Always (4)”. The scale scores range from a minimum of 16 to a maximum of 64, with higher scores indicating higher levels of parental stress. The psychometric analysis demonstrated high internal consistency, yielding a Cronbach’s alpha value of 0.85 and a Spearman-Brown split-half reliability of 0.82. In the present study, Cronbach’s alpha for the PSS was 0.821, indicating strong internal consistency within our sample.

#### Quality of life questionnaire in autism - parent version (QoLA)

The QoLA, originally developed by Eapen *et al*,^39^ was adapted into Turkish by Özgür *et al*^40^ to assess the quality of life of parents with children diagnosed with ASD. The questionnaire consists of two distinct parts, both using a 5-point Likert scale. Part A contains 28 items measuring the parents’ perception of their own quality of life, rated from 1 (‘Not at all’) to 5 (‘Very much’). Part B consists of 20 items assessing the extent to which the child’s autism-specific behaviours disrupt the parents’ daily lives. In Part B, a score of 5 indicates the behaviour is ‘not a problem at all’. Thus, higher scores in Part B reflect fewer problems and a lower perceived autism-related burden. The Turkish adaptation identified six sub-dimensions for Part A and three for Part B through exploratory factor analysis. The instrument demonstrates high internal consistency, with Cronbach’s alpha coefficients of 0.928 for Part A and 0.944 for Part B. For the current sample, the Cronbach’s alpha coefficients were 0.922 for Part A and 0.832 for Part B, indicating excellent reliability.

### Statistical analysis

All data were analysed using SPSS V.22.0. Data collection yielded a total of 111 fully completed and valid questionnaires, which constituted the final sample for the analyses. Normality of continuous variables was assessed using the Shapiro-Wilk test. Normally distributed data were expressed as mean±standard deviation (X±SD). Categorical variables were presented as frequencies (n) and percentages (%).

The analytic strategy was structured to test the study’s three primary hypotheses:

1. To address H1 and H2, bivariate associations between children’s mealtime behaviours (BAMBI), PMAS, parental stress (PSS) and quality of life (QoLA-B) were assessed using Pearson’s correlation coefficient.
2. To test H3, a multivariate linear regression analysis was conducted to identify independent predictors of diet quality (HEI-2020). In this model, BAMBI scores and PMAS were entered as independent variables, while child characteristics (age, BMI-Z score) and household income were included as covariates to account for potential confounding.
3. Group comparisons (eg, gender-based differences) were performed using the independent samples t-test for normally distributed variables or the Mann-Whitney U test for non-normal distributions.

For group comparisons, the independent samples t-test was used for normally distributed variables, while the Mann-Whitney U test was employed for non-normal distributions. Gender comparisons were conducted despite unequal group sizes (Boys n=85 vs Girls n=26), as this distribution reflects the known male preponderance in ASD prevalence. Non-parametric tests were prioritised to account for potential variance differences arising from unequal sample sizes. Effect sizes were reported as Cohen’s d for parametric tests and rank-biserial correlation (r) for non-parametric tests. The χ^2^ test was used for categorical comparisons. Correlations between continuous variables were assessed using Pearson’s correlation coefficient (r). A p value of <0.05 was considered statistically significant.

Following the correlation analysis, a multivariate linear regression analysis was conducted to identify the independent predictors of diet quality (HEI-2020). The model included children’s mealtime behaviours (BAMBI) and PMAS as the primary independent variables, while adjusting for key sociodemographic covariates: child’s age, BMI-z score and household income. To ensure model stability and prevent overfitting, other psychosocial variables were evaluated via bivariate correlations. The validity of the regression model was rigorously verified against fundamental assumptions; multicollinearity was ruled out as Variance Inflation Factor values for all predictors were below 1.25. The normality of residuals was confirmed by the Shapiro-Wilk test, and homoscedasticity was verified through the Breusch-Pagan test. Results were evaluated using R^2^, standardised regression coefficients (β) and significance levels (p<0.05).

In addition to numerical analyses, data visualisation techniques were employed to further illustrate the clinical impact of feeding challenges on nutritional and psychosocial outcomes. To achieve this, the sample was stratified into ‘Low BAMBI’ and ‘High BAMBI’ groups based on the median score of problematic mealtime behaviours. Boxplots were generated using the *ggplot2* package within the R programming environment to visually compare these two groups across key study variables, including diet quality (HEI-2020), PSS, parental feeding strategies (PMAS) and quality of life (QoLA-Part A and Part B). These visualisations display the medians, IQRs and individual jittered data points, providing a clear graphical representation of the group differences.

### Reporting guideline

This study was designed and reported in accordance with the Strengthening the Reporting of Observational Studies in Epidemiology (STROBE) statement for cross-sectional studies.

## Results

### Sociodemographic and clinical characteristics

A total of 111 parents, who met all inclusion criteria and provided written informed consent, were enrolled in the study from two special education and rehabilitation centres in Antalya, Türkiye, during the data collection period (1 September 2022 to 1 December 2022). The sociodemographic characteristics of the children and their families are presented in table 2. The mean age of the children was 6.8 ± 3.5 years, and the majority of the sample consisted of boys (76.6%). The mean maternal and paternal ages were 36.5 ± 7.0 years and 41.1 ± 8.4 years, respectively. Among the mothers, 30.6% were university graduates, while 40.5% of the fathers had completed high school. More than half of the families (52.3%) reported that their household expenses exceeded their income. Additionally, 13.5% of the participants stated that they had another family member or sibling diagnosed with ASD.

**Table 2:** Evaluation of demographic characteristics of children and families

Variables	Boys (n=85)	Girls (n=26)	Total (n=111)	P value
	X±SD	X±SD	X±SD	
Age (year)	7.1±3.4	5.6±3.4	6.8±3.5	**0.017**
Mother’s age (year)	36.6±7.1	36.1±6.8	36.5±7	0.898
Father’s age (year)	41.5±8.6	39.6±7.4	41.1±8.4	0.690
Number of children had	2.0±0.8	2.6±1.2	2.2±1	0.068
Mother’s education level	**n (%)**	**n (%)**	**n (%)**	**X** ^**2**^ **; P value**
Illiterate	2 (2.4)	2 (7.7)	4 (3.6)	0.392*
Primary school	20 (23.5)	7 (26.9)	27 (24.3)	
Middle school	11 (12.9)	5 (19.2)	16 (14.4)	
High school	23 (27.1)	7 (26.9)	30 (27.0)	
University	29 (34.1)	5 (19.2)	34 (30.6)	
Father’s education level	**n (%)**	**n (%)**	**n (%)**	**X^2^; P value**
Illiterate	1 (1.2)	2 (7.7)	3 (2.7)	0.280*
Primary school	18 (21.2)	5 (19.2)	23 (20.7)	
Middle school	12 (14.1)	2 (7.7)	14 (12.6)	
High school	32 (37.6)	13 (50.0)	45 (40.5)	
University	21 (24.7)	4 (15.4)	25 (22.5)	
Family income level	**n (%)**	**n (%)**	**n (%)**	**X^2^; P value**
Income more than expenses	6 (7.1)	4 (15.4)	10 (9.0)	2.285; 0.321
Income equal to expenses	29 (34.1)	10 (38.4)	39 (35.1)	
Income less than expenses	47 (55.3)	11 (96.2)	58 (52.3)	

Bold entries indicate statistically significant results (p<0.05).

* Fisher’s exact test.

Detailed findings related to children’s characteristics are shown in table 1. The mean age remained consistent across analyses (6.8 ± 3.5 years, p>0.05). Regarding height status, 45.9% of the children were classified as short for their age, 35.1% had normal height and 18.9% were tall. According to the WHO BMI-for-age classification, the mean BMI Z-score of the participants was 1.15±2.45. Regarding the BMI categories, 3.6% (n=4) were underweight, 53.2% (n=59) had a normal weight and 43.2% (n=48) were overweight or obese (16.2% overweight and 27.0% obese). There were no significant differences between genders in terms of mean BMI Z-scores (p=0.253) or BMI categories (p=0.244). Notably, 70.3% of children were reported as picky eaters, and 29.7% were reported to skip main meals. Food allergies were present in 9.9% of the sample. Breastfeeding prevalence was high, with 96.4% of children having been breastfed. Regarding physiological feeding-related complications, 19.8% of the children were reported to have chewing and swallowing difficulties, and 29.7% experienced constipation. Additionally, 8.1% had diarrhoea and 4.5% had documented food intolerances. Parents were queried regarding the presence of accompanying chronic medical or psychiatric diagnoses. The majority of children (82.9%) had no reported comorbidities. The most common medical comorbidity was epilepsy (6.3%). While formal psychiatric diagnoses such as Attention-Deficit/Hyperactivity Disorder (ADHD) or Generalised Anxiety Disorder (GAD) were not reported in the medical history, behavioural manifestations associated with these conditions were prevalent; 56.8% of the children exhibited hyperactivity and 27.0% displayed temper tantrums (table 1).

### Child feeding behaviours and diet quality

The evaluation of diet quality and mealtime behaviours is summarised in table 3. Diet quality, assessed using the HEI-2020, yielded a mean score of 34.4 ± 10.6, indicating that the majority of children (94.5%) had diets classified as “poor” (table 3). Mealtime behaviours were evaluated using the BAMBI. The mean BAMBI total score was 42.7 ± 10.7. PMAS revealed that the most frequently employed practices were offering multiple food options (mean: 8.2 ± 1.9) and positive persuasion (mean: 9.2 ± 2.9).

**Table 3:** Evaluation of children’s diet quality and meal behaviours, and parental eating time behaviours, stress levels, and quality of life

	Boys x±SD	Girls x±SD	Total x±SD	P value
HEI score	35.3±10.8	31.2±9.5	34.4±10.6	0.082
HEI classification	**n (%**)	**n (%**)	**n (%**)	**x^2^; P value**
Poor	78 (92.9)	26 (100.0)	104 (94.5)	0.332*
Needs improvement	6 (7.1)	–	6 (5.5)	
Good	–	–	–	
	**x±SD**	**x±SD**	**x±SD**	**P value**
BAMBI Total	41.6±10.2	46.5±11.4	42.7±10.7	0.061
Food Refusal	9.2±3.8	10.8±4.7	9.5±4.1	0.069
Restricted Food Choice	22.5±6.6	24.1±5.5	22.8±6.4	0.267
Autism-Related Behaviours	10.2±4.1	11.6±4.0	10.6±4.1	0.130
PMAS	62.3±6.3	62.7±7.6	62.4±6.6	0.764
Snacking Amount	7.9±1.6	7.6±1.9	7.9±1.7	0.402
Positive Persuasion	9.2±2.9	8.8±3	9.2±2.9	0.631
Daily Fruit Vegetable Options	7.7±1.1	7.6±1.4	7.7±1.2	0.665
Use of Rewards	7.3±2.2	8.2±2.1	7.5±2.2	0.081
Insistence on Eating	3.9±1.6	4.4±1.7	4±1.6	0.061
Snacking Pattern	5.6±1.5	5.5±1.1	5.6±1.4	0.402
Special Meals	6.2±1.9	6.3±1.5	6.2±1.8	0.657
Reduction of Animal Fats	6.4±1.3	6.3±1.5	6.4±1.3	0.738
Multiple Meal Options	8.2±2	8.2±1.6	8.2±1.9	0.827
PSS	32.4±9.7	37.7±8.4	33.6±9.7	**0.017 (r=0.22)**
QoLA				
A-Parent Quality of Life	98.8±21	91.3±23.4	97.1±21.7	0.124
B-Parental Perception of Difficulty	62.5±16.4	51.6±15	59.9±16.7	**0.003 (d=0.68)**

Effect sizes are reported for statistically significant group comparisons: r=rank-biserial correlation for Mann-Whitney U test, d=Cohen’s d for t-test. Bold entries indicate statistically significant results (p<0.05).

* Fisher’s exact.

BAMBI, Brief Autism Mealtime Behaviour Inventory; HEI, Healthy Eating Index; PMAS, Parental Mealtime Strategies; PSS, parental stress scale; QoLA, Quality of life questionnaire in autism.

### Parental psychosocial outcomes and mealtime strategies

The mean total score on the PSS was 33.6 ± 9.7, reflecting moderate stress levels among participating parents. QoLA questionnaire showed that parents’ perceived quality of life (section A) had a mean score of 97.1 ± 21.7, while their perceived burden due to their child’s autism-specific difficulties (section B) averaged 59.9 ± 16.7 (table 3). Caregivers of girls reported significantly higher stress levels compared with caregivers of boys (p=0.017, r=0.22), indicating a small-to-medium effect size. Similarly, the perceived burden of perceived autism-related burden (QoLA-B) was significantly higher in families of girls compared with boys (t(109)=−3.03, p=0.003, d=0.68), representing a medium-to-large effect size.

### Correlation matrix of the study variables

Bivariate correlation analyses are summarised in table 4. A significant negative correlation was found between BAMBI scores and HEI scores (r = –0.302, p = 0.002), indicating that more severe mealtime behaviour problems were associated with poorer diet quality. Moreover, significant correlations were observed between parental feeding practices such as the amount of snacking, positive persuasion and the preparation of special meals.

**Table 4:** Evaluation of the relationship between children’s meal behaviours, parental eating time behaviours, stress levels and quality of life with HEI-2020

	HEI-2020					
	Girls		Boys		Total	
	r	P value	r	P value	r	P value
BAMBI	−0.263	0.214	−0.262	**0.016**	−0.302	**0.002**
Restricted food selection	−0.301	0.153	−0.256	0.019	−0.304	**<0.001**
Food Refusal	−0.216	0.312	−0.151	0.170	−0.192	**0.047**
Autism-specific behaviours	−0.135	0.530	0.000	0.998	−0.056	0.566
PMAS						
Snacking amount	−0.165	0.421	0.293	**0.007**	0.199	**0.037**
Positive persuasion	0.090	0.662	0.126	0.255	0.120	0.213
Daily fruit and vegetable	0.239	0.239	0.145	0.189	0.148	0.123
Use of rewards	−0.022	0.915	−0.114	0.302	−0.132	0.168
Insistence on eating	0.186	0.363	0.166	0.132	0.115	0.233
Snacking pattern	−0.109	0.597	−0.070	0.528	−0.079	0.414
Special meals	−0.149	0.466	−0.196	0.075	−0.210	**0.028**
Reduction of animal fats	0.195	0.340	0.039	0.726	0.068	0.478
Multiple meal options	0.130	0.526	0.013	0.904	0.016	0.869
Stress Scale	−0.150	0.464	−0.033	0.766	−0.081	0.400
Quality of Life						
A	0.035	0.864	−0.019	0.867	0.013	0.886
B	0.113	0.584	0.575	**0.019**	0.104	0.278

Bold entries indicate statistically significant results (p<0.05).

BAMBI, Brief Autism Mealtime Behaviour Inventory; HEI, Healthy Eating Index; PMAS, Parental Mealtime Strategies.

Furthermore, supporting our second hypothesis (H2), a significant negative correlation was found between parental stress (PSS) and perceived autism-related burden (QoLA-Part B) (r=−0.424, p<0.001) (Data not shown).

Even after controlling for sociodemographic factors, BAMBI remained a strong and significant negative predictor of diet quality (B=−0.370, β=−0.373, p<0.001) (table 5). The overall model was statistically significant (F (5, 98)=3.424, p=0.007), explaining 14.9% of the variance in diet quality (R^2^=0.149). Furthermore, PMAS emerged as a significant independent positive predictor of HEI-2020 scores (B=0.387, β=0.245, p=0.015), confirming that parental interventions independently influence dietary outcomes. Other covariates, including age, household income, and BMI-Z score, did not show statistically significant associations in this model (p>0.05) (table 5).

**Table 5:** Multivariate linear regression analysis identifying independent predictors of diet quality (Healthy Eating Index-2020) in children with Autism Spectrum Disorder

Variables	B	SE	β	t	P value	95% CI for B
Constant	25.538	9.958		2.565	0.012	(5.776 to 45.299)
Child’s age	0.015	0.330	0.005	0.046	0.964	(−0.64 to 0.67)
BMI-Z Score	−0.134	0.404	−0.031	−0.332	0.740	(−0.935 to 0.667)
Household income*	2.074	3.826	0.055	0.542	0.589	(−5.519 to 9.666)
BAMBI	−0.370	0.104	−0.373	−3.561	**<0.001**	(−0.577 to −0.164)
PMAS	0.387	0.156	0.245	2.481	**0.015**	(0.077 to 0.697)

Bold entries indicate statistically significant results (p<0.05).

* Household income was coded as binary (0: Income greater than or equal to Expenses, 1: Income less than Expenses). Model summary: R2=0.149; Adjusted R2=0.105; F (5, 98)=3.425; p=0.007.

BAMBI, Brief Autism Mealtime Behaviour Inventory; Beta, Standardised Beta Coefficient; BMI, Body Mass Index; PMAS, Parental Mealtime Strategies.

**Figure 1:**
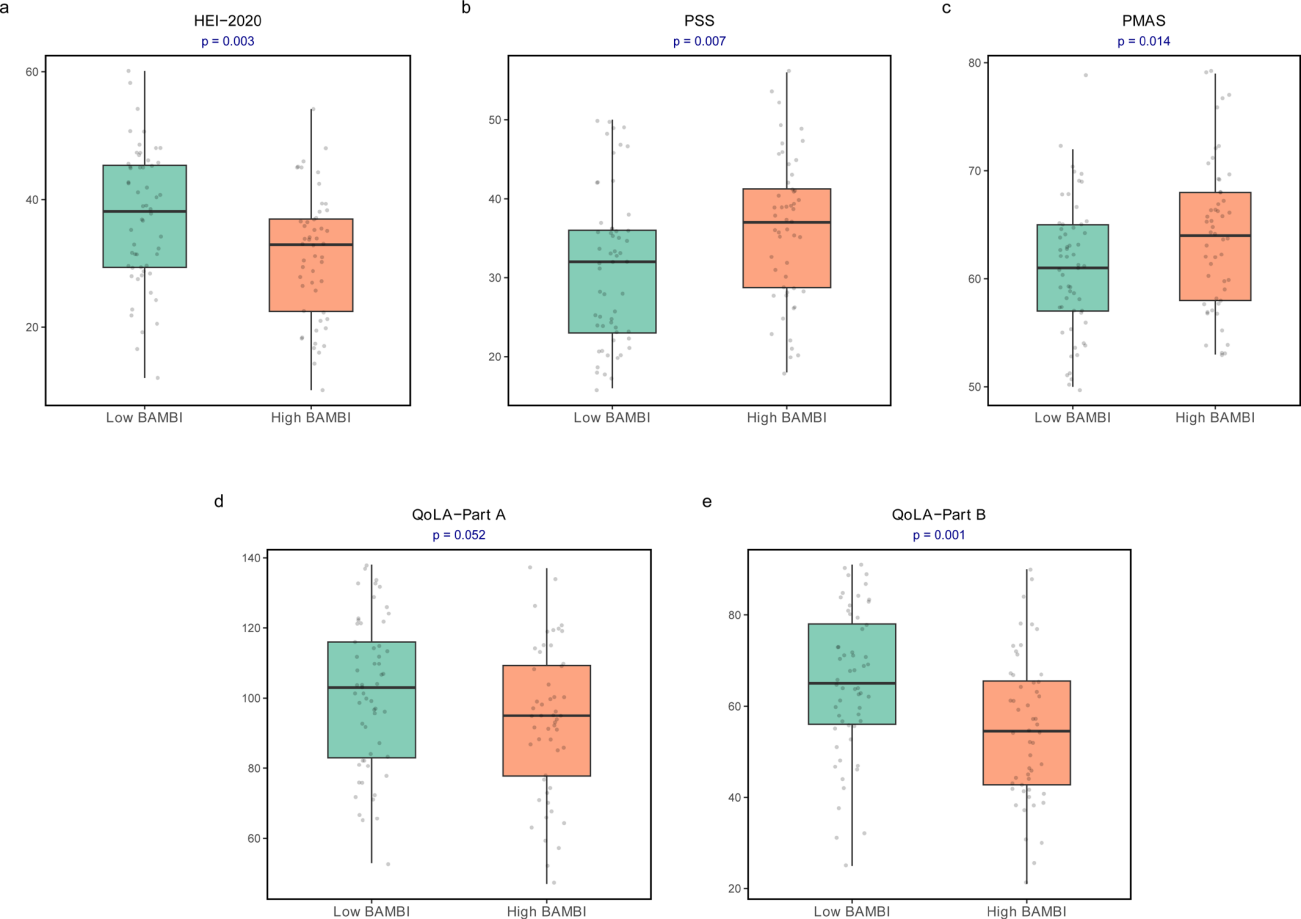
Comparison of nutritional and psychosocial outcomes by mealtime behaviour severity. Groups were stratified based on the median BAMBI score (median=42) into Low BAMBI and High BAMBI groups to better illustrate clinical differences. Boxplots represent: (**a**) Diet Quality (HEI-2020), (**b**) Parental stress (PSS), (**c**) Parental Mealtime Strategies (PMAS), (**d**) General Parental Quality of Life (QoLA-Part A) and (**e**) Autism-Related Burden (QoLA-Part B). The horizontal lines within the boxes represent the medians, and the whiskers extend to the 1.5x IQR. Individual data points are displayed as jittered dots. Blue-teal boxes indicate the Low BAMBI group, and orange-salmon boxes indicate the High BAMBI group. P values represent the results of independent samples t-tests. BAMBI, brief autism mealtime behaviour inventory; HEI, Healthy Eating Index.

To provide a more clinically relevant perspective on the impact of feeding challenges, as gender-based differences were found to be less influential than clinical severity, the study sample was stratified into ‘Low BAMBI’ and ‘High BAMBI’ groups based on the median score of problematic mealtime behaviours (figure 1). This categorisation allows for a clearer visualisation of how the intensity of behavioural dysregulation independently relates to nutritional and psychosocial outcomes.

Significant clinical differences were observed across all primary and secondary outcomes when stratified by mealtime behaviour severity (figure 1). As demonstrated in figure 1a, children in the High BAMBI group exhibited significantly lower diet quality (HEI-2020) compared with those in the Low BAMBI group. Parallel to the decline in child nutrition, parents of children with higher behavioural challenges reported markedly higher levels of parental stress (PSS) (figure 1b) and a greater frequency of used mealtime strategies (PMAS) (figure 1c). Furthermore, the psychosocial impact was more pronounced in autism-specific domains, while general quality of life (QoLA-Part A) did not differ significantly between groups (figure 1d), parents in the High BAMBI group reported a significantly higher autism-related perceived burden (QoLA-Part B) (figure 1e).

## Discussion

Feeding difficulties in children with ASD represent a critical area of concern, with significant implications not only for nutritional adequacy but also for family dynamics and parental well-being.^28 41^ This study, conducted in Türkiye, explored the associations among mealtime behaviours, diet quality, and caregiver psychosocial outcomes in families of children with ASD. Specifically, our results demonstrate that higher levels of mealtime behavioural disturbances are directly associated with a significant decline in children’s diet quality, as measured by the HEI-2020. To further refine these observations and address the clinical heterogeneity within the sample, we examined these relationships by stratifying participants based on the median BAMBI score. This analysis revealed that the impact of feeding challenges is not uniformly distributed across all domains of life quality. While child diet quality and autism-related parental burden showed significant sensitivity to increased behavioural severity, general parental well-being remained relatively stable. These results suggest that the psychosocial toll of feeding difficulties may be context-specific, primarily targeting the perceived burden of autism-related care rather than broader social or economic aspects of life quality.

A major finding was the high prevalence of poor diet quality among children with ASD, with 94.5% of the sample scoring in the poor category on the HEI-2020. While some studies suggest that diet quality in ASD may be comparable to that of typically developing children,^42 43^ our findings challenge these assumptions. It is important to note that the HEI-2020 is used here as a comparative rather than a normative indicator, as composite indices may not fully capture culturally specific dietary patterns or the clinical nuances of nutrient inadequacies in non-U.S. contexts. The utilisation of the HEI-2020 in this study is justified by the specific nature of nutritional risk in ASD, where the primary concern is often not total energy intake but a highly restricted dietary pattern. Recent meta-analyses confirm that while children with ASD may not show significant differences in total calorie intake, they exhibit significantly lower intake of proteins and essential lipid-soluble (A, D, K) and water-soluble (folate, riboflavin) vitamins compared with typically developing peers, leading to higher risks of malnutrition and impaired physical development.^44 45^ By assessing dietary quality through standardised patterns rather than isolated nutrient metrics, we provide a more comprehensive reflection of the food selectivity and ‘malnutrition susceptibility’ that characterise this population.^45^ This approach also addresses the lack of standardised ASD-specific tools by employing a validated index that captures the clinical reality of dietary rigidity.^46 47^ Rather than relying solely on descriptive parental reports of picky eating, our multivariate linear regression analysis provides robust quantitative evidence for this dietary deterioration. Even after adjusting for the child’s age, BMI-z score and household income, the severity of mealtime behavioural problems emerged as a highly significant independent predictor of poorer diet quality. This demonstrates that the profoundly low HEI scores are directly driven by the core rigidities and sensory-based food refusals inherent to ASD,^48 49^ systematically narrowing the child’s food repertoire to ultra-processed or nutrient-poor options.

Notably, a strong inverse association was found between BAMBI scores and HEI scores, suggesting that increased mealtime behaviour problems are strongly correlated with lower diet quality. While the multivariate model identified BAMBI and PMAS as a significant predictor, it accounted for a modest 14.9% of the total variance. This suggests that diet quality in children with ASD is a complex, multifactorial outcome shaped by neurodevelopmental severity, sensory sensitivities, gastrointestinal comorbidities and family routines. Consequently, our findings should be interpreted as exploratory associations rather than robust predictive pathways, as behavioural predictors must be situated within this broader clinical and contextual framework to be meaningfully interpreted. This behavioural impact aligns with studies emphasising how disruptive behaviours like refusal to sit, tantrums and rituals compromise dietary adequacy.^29^ Our results further concur with Omara *et al*^50^ who observed that severe ASD is linked to lower diet quality through limited food selectivity. Specifically, sensory-based problems like the preference for crunchy or only sweet foods reinforce the rejection of balanced food groups, supporting our low HEI scores. This association likely reflects a bidirectional and transactional process between child behaviour and parental responses. While accommodative parental strategies may correlate with poorer diet quality, these practices are often a reactive response to the stress of persistent food refusal and behavioural rigidity in children with ASD. Rather than a simple causal pathway, our findings suggest a complex feedback loop where child-led feeding challenges and parental coping mechanisms continuously influence one another. Given the cross-sectional nature of our study, these findings highlight important clinical associations rather than direct causal pathways.

Interestingly, our multivariate model revealed that sociodemographic factors such as household income and the child’s age did not significantly predict diet quality when behavioural problems were accounted for. This suggests that the impact of problematic mealtime behaviours on nutritional intake is pervasive and robust, overriding the potential protective effects of higher socioeconomic status or developmental maturation. Thus, behavioural dysregulation during meals can be considered both a cause and consequence of poor diet quality. Our findings map out a clear transactional pathway: severe mealtime behavioural dysregulation fundamentally compromises the child’s nutritional intake, which in turn deeply disrupts daily family routines and drives up the caregiver’s perceived burden. It is this cumulative psychosocial load and daily disruption rather than just the isolated clinical diagnosis of the child that ultimately dictates parental stress levels. This interpretation aligns with transdiagnostic and family-centred models, which emphasise that the psychosocial load for caregivers stems from the broader impact of the disorder on family functioning. Consistent with international literature, such as studies by Garrido *et al*,^51^ John *et al*^30^ and Konowałek *et al*,^52^ our results confirm that perceived psychosocial burden—rather than objective clinical symptoms—is the primary determinant of parental quality of life. These findings underscore the need for integrated, family-centred interventions that address the caregiver’s overall psychological well-being alongside the child’s behavioural challenges.

Consistent with our third hypothesis, PMAS emerged as a significant independent predictor of children’s diet quality. Interestingly, our multivariate regression model revealed that the total PMAS score had a significant positive effect on HEI-2020 scores. This suggests that structured and proactive parental interventions—such as ensuring daily fruit and vegetable availability or using positive persuasion—serve as protective factors that enhance overall diet quality. However, bivariate analyses highlighted the ‘double-edged’ nature of parental strategies: specific accommodative practices, such as frequently preparing special meals, were negatively correlated with diet quality. While preparing a child’s preferred foods may function as a necessary short-term coping mechanism to ensure caloric intake in the face of severe food refusal, from a behavioural standpoint, this accommodation acts as an inadvertent reinforcement. Over time, this permissive loop perpetuates food selectivity, narrows the dietary repertoire, and ultimately drives the diet quality down. Therefore, interventions should empower parents to transition from reactive accommodations (eg, special meals) to proactive, structured feeding routines.

Parental distress during feeding is not a trivial issue; it can impair compliance with treatment, exacerbate child behavioural rigidity, and diminish the emotional availability needed to support food-related learning.^53^ Thus, improving caregiver coping resources must be a central component of intervention design. These findings emphasise the necessity of going beyond traditional dietary education in ASD populations. Multidisciplinary feeding interventions that include dietitians, behavioural therapists and occupational therapists have shown promise in increasing food acceptance and reducing mealtime stress. Moreover, parent-mediated approaches, such as food chaining, systematic desensitisation and differential reinforcement, can be effective in broadening food repertoires while simultaneously empowering caregivers. Importantly, integrating stress management components into such programmes may further enhance intervention efficacy and caregiver resilience. Based on these findings, we recommend a shift from isolated dietary advice to comprehensive, multidisciplinary clinical protocols. First, clinicians should routinely screen children with ASD for ARFID using DSM-5 criteria to identify those requiring intensive medical monitoring. Second, interventions should employ structured, evidence-based strategies such as ‘Food Chaining’ or the Sequential Oral Sensory (SOS) approach,^54^ rather than coercive feeding practices. Finally, given the strong link between parental burden and child outcomes, caregiver stress management (eg, cognitive-behavioural support) must be integrated into the standard of care for PFDs in ASD.

Although income level was overshadowed by behavioural factors in our predictive model, the financial strain experienced by these families cannot be overlooked. More than half of the families in our study stated that they spent more than they earned, which may restrict access to high-quality foods, nutritional counselling or private therapy services. Financial stress may also compound psychological distress, further limiting adaptive capacity. Previous research showed that lower parental education and income are associated with worse dietary and psychological outcomes in ASD.^55 56^ Support systems must be designed with these inequities in mind, ensuring that interventions are not only effective but also accessible and affordable.

A striking finding of this study is the pronounced psychosocial dysfunction observed in the families of girls with ASD. Despite the established male preponderance in our sample, reflecting typical ASD prevalence rates, caregivers of girls reported significantly higher levels of parenting stress and a much heavier perceived burden of autism-related difficulties compared with caregivers of boys. Given the unbalanced gender distribution (85 boys vs 26 girls) and the significant age differences between these groups, these results should be viewed as exploratory and hypothesis-generating rather than definitive. Rather than reflecting objective differences in symptom severity, these findings likely represent a complex interplay of sex-specific caregiver interpretations and societal norms. Consequently, when feeding difficulties manifest in girls with ASD, they trigger a profoundly heavier psychosocial load, frustration, and isolation for the parents. Future research with gender-matched groups is necessary to clarify whether these perceptions stem from distinct phenotypic profiles or varying psychosocial pressures associated with raising girls with ASD.

This study has several limitations that warrant consideration. First, the cross-sectional design precludes causal inferences regarding the bidirectional relationship between parental stress and child feeding behaviours. Second, although all participants had a clinically verified ASD diagnosis, the absence of data on ASD severity levels and intellectual disability limits our ability to identify whether specific feeding phenotypes are more prevalent in certain ASD subgroups. Future research should use phenotypically stratified cohorts to better understand the nuances of diet quality across the autism spectrum. Third, while the study covered a broad age range, the sample was predominantly composed of children in early-to-middle childhood, with insufficient statistical power to conduct valid subgroup analyses for adolescents. Fourth, regarding clinical history, we lacked detailed data on the type and intensity of concurrent non-dietary therapies (eg, weekly hours of ABA or sensory integration), limiting our control over their potential therapeutic effects. Finally, a formal recruitment log was not maintained, preventing the calculation of a precise response rate, and the study did not include a measure of social validity to assess parents’ perceived feasibility of the feeding strategies. Future longitudinal studies with stratified samples and mixed-methods designs are needed to address these gaps.

This study contributes to the growing body of evidence on the bidirectional relationship between child feeding behaviours and parental psychological well-being in families affected by ASD. The findings of this study demonstrate a significant, multidimensional association between problematic mealtime behaviours, compromised diet quality, and elevated parental psychosocial stress in children with ASD. These results highlight that feeding challenges in this population are not isolated nutritional issues but are deeply intertwined with family dynamics and caregiver well-being. Socioeconomic disparities and gendered parental expectations further shaped these dynamics. Crucially, our findings indicate that behavioural rigidity affects diet quality independently of the child’s developmental stage, suggesting that waiting for children to ‘outgrow’ these behaviours is not a viable strategy. While our findings are consistent with the international literature, cultural factors unique to Türkiye, such as family mealtime norms and gender roles, may influence how feeding difficulties are perceived and managed. Therefore, future interventions should adopt a biopsychosocial, family-centred approach that combines structured feeding support with caregiver mental health strategies. Recognising and addressing both the nutritional and emotional needs of children and their caregivers is essential for improving long-term outcomes in this population.

## Data Availability

No data are available.
