# Supplementary material for: Mealtime behaviours, diet quality and parental stress in children with autism spectrum disorder: a cross-sectional study from Türkiye
Source: BMJ Paediatr Open. 2026 Jun 23;10(1):e004226. doi: 10.1136/bmjpo-2025-004226 (PMC13295970; doi:10.1136/bmjpo-2025-004226)
Supplement: Supplementary file 1 [file bmjpo-2025-004226.reviewer_comments.pdf]

PEER REVIEW HISTORY

BMJ Paediatrics Open publishes all reviews undertaken for accepted manuscripts. Reviewers are asked to complete a checklist review form and are provided with free text boxes to elaborate on their assessment. These free text comments are reproduced below.

ARTICLE DETAILS

**TITLE (PROVISIONAL)**	MEALTIME BEHAVIORS, DIET QUALITY AND PARENTAL STRESS IN CHILDREN WITH AUTISM SPECTRUM DISORDER: A CROSS-SECTIONAL STUDY FROM TÜRKİYE
**AUTHORS**	OZYILDIRIM, CANER<br>KARACIL ERMUMCU, Merve Seyda<br>MUTLU, Meryem Ece<br>BARAN, Ilgın<br>SALİM, Hediye<br>SEREMET KURKLU, Nilgun

VERSION 1 – REVIEW

**REVIEWER NAME**	Phalen, James
**REVIEWER AFFILIATION**	
**REVIEWER CONFLICT OF INTEREST**	No Conflict of Interest
**AI DISCLOSURE**	No, I have not used AI
**DATE REVIEW RETURNED**	17-Nov-2025

**GENERAL COMMENTS**	The authors tackle a problem commonly found in children who have ASD: picky eating or food selectivity. While this topic is relevant, the manuscript have several weaknesses which the authors must address.<br><br>Foremost, they assume the diagnosis of ASD for 111 patient but fail to document how or by whom the diagnosis was made. The reference standard is a qualified diagnostician administering an appropriate module of the Autism Diagnostic Observation Schedule, Second Edition (ADOS-2) along with standardized testing of cognitive or intelligence skills, and review of the DSM-5-TR diagnostic criteria. Withing this context, the author must then differentiate between patients which ADOS-2 substantiated ASD with average intelligence versus those who have intellectual disability (ID) (i.e., a cognitive developmental quotient (DQ) or an IQ < 70.<br><br>Did the patients have any comorbidities (e.g., attention-deficit/hyperactivity disorder [ADHD], generalized anxiety disorder [GAD], etc.)? The authors must address this.<br><br>The authors use broad, non-specific terminology of feeding problems, picky eating, and food selectivity. These are common in neurotypical children, affecting up to 40% of them. The authors must instead use feeding-related diagnoses such as pediatric feeding disorder (PFD) or, possibly, avoidant/restrictive food intake disorder (ARFID), both of which have diagnostic criteria and ICD-10 codes associated with them.

	Did any of the patients have feeding-related complications (e.g., oropharyngeal dysphagia, chronic constipation, severe gastroesophagel reflux disease [GERD])? Each of these can affect a child's food intake.<br><br>Although, while the author's intention and the tone of the manuscript are commendable, the study as written lacks the scientific rigor needed to make it worthy of publication at this time.

**REVIEWER NAME**	Esposito, Marco
**REVIEWER AFFILIATION**	Dep. Life of Sciences
**REVIEWER CONFLICT OF INTEREST**	No Conflict of Interest
**AI DISCLOSURE**	Grammarly for no native speaker
**DATE REVIEW RETURNED**	25-Nov-2025

**GENERAL COMMENTS**	Thank you for inviting me to revise the following manuscript: Mealtime Behaviors, Diet Quality, and Parental Stress in Children with Autism Spectrum Disorder: A Cross-Sectional Study from Türkiye.<br>Journal: BMJ Paediatrics Open; Manuscript ID: bmjpo-2025-004226<br><br>This manuscript addresses a significant and clinically relevant topic: the intersection between feeding difficulties, diet quality, and parental stress in children with Autism Spectrum Disorder (ASD). The study is clearly motivated, the sample is well defined, and the use of validated psychometric instruments is commendable. The manuscript has several strengths, appropriate ethical procedures, and a meaningful discussion that attempts to contextualize the findings within the broader ASD and feeding-behavior literature. However, several methodological, reporting, and interpretative issues require substantial revision. Some sections lack conceptual precision, confident design choices are not sufficiently justified, and key variables are missing or insufficiently described. Addressing these concerns will significantly enhance the rigor, clarity, and interpretability of the study.<br><br>I have carefully revised the manuscript point by point.<br><br>INTRODUCTION<br>This section lacks methodological detail in the cited studies, including sample characteristics, methodology, primary findings, and contextual relevance to the present study. This omission weakens the rationale. For example, describing sample sizes, age ranges, the types of measures used, and core results provides essential grounding and allows the reader to understand how the present work advances the field (by connecting previous studies with the current one). Furthermore, no references to the AAP Pediatric Nutrition Guidelines, CDC ASD feeding resources, or NICE ASD guidelines would enhance clinical relevance. Generally, the previous studies and a primary updated literature are recommended.<br><br>Moreover, research questions and hypotheses are not explicitly stated. The manuscript states a general aim but does not articulate specific, testable hypotheses. Explicit hypotheses would improve coherence and allow more precise evaluation of the results, such as H1: Higher selective behaviors scores are associated with lower HEI scores. H2: Greater parental stress is associated with higher

	perceived burden. H3: Parental mealtime strategies (e.g., special meals) predict variations in diet quality. This would align well with the analyses already conducted.<br><br>METHOD<br>I noted missing details on recruitment and participant flow. While the manuscript states that participants were recruited from two educational centers, several key details are missing: the number of parents approached, the number who declined, and whether consecutive sampling included all families. Any inclusion criteria regarding ASD diagnosis certification, whether children were attending behavioral, nutritional, or therapeutic interventions. These omissions limit the evaluation of selection bias. A flow diagram would be helpful, given the extensive age range and the lack of subgroup analyses. The sample ranges from 2 to 19 years, which is developmentally heterogeneous for feeding behavior research. Yet no subgroup or cluster analyses (e.g., toddlers vs. school-age vs. adolescents) were conducted, nor was a rationale provided for treating the group as homogeneous. Mainly, age strongly influences eating autonomy, sensory development, parental feeding strategies, BMI, and HEI comparability. The current research design seems weak (without comparison and models).<br><br>The manuscript reports Cronbach's α values and cites Turkish adaptations. However, key information missing includes: item response formats (e.g., 1–5 Likert scale), interpretative cut-offs (if any), and scoring rules (sum vs. mean; handling missing items). Including this information increases transparency and reproducibility. The instruments are too synthetic, lacking examples and prior applications.<br><br>The analytical approach is too simplistic; some points need refinement: Pearson vs Spearman correlations, HEI, and BAMBI scores often show non-normal distributions in ASD samples. If normality were violated, Spearman correlations would be more appropriate. Given socioeconomic variables influenced outcomes, a model would more convincingly establish associations while controlling for confounders (income, age, BMI, sex). No reporting of effect sizes: T-tests and Mann–Whitney U tests should include: Cohen's d, r effect size, and confidence intervals. No correlation or exploratory models are applied. Also, the manuscript does not report: ASD severity, cognitive level, communication abilities, comorbidities beyond brief mention, type or intensity of therapy (ABA, sensory integration, feeding therapy). These variables are directly related to feeding behavior and stress, and their omission may lead to misinterpretation.<br><br>RESULTS<br>The Results section is generally clear and presents substantial descriptive and inferential data; however, several important issues require clarification or refinement to strengthen transparency, coherence, and interpretability. The section provides extensive raw descriptive data (e.g., anthropometrics, feeding behaviors, dietary habits, socioeconomic variables), but the narrative lacks a clear structure. The reader would benefit from clearer organization, including sections on child characteristics, feeding-related variables, parental outcomes, and the associations among variables. This would improve readability and conceptual coherence.<br><br>Moreover, the results do not include several essential variables that

	are typically necessary to interpret feeding behaviors in ASD, such as ASD severity levels, cognitive or adaptive functioning, the type or intensity of interventions (e.g., ABA, sensory integration, feeding therapy), communication level, and comorbidities beyond broad categories. The absence of these data limits the capacity to understand heterogeneity within the sample and may confound observed associations. At a minimum, this limitation should be acknowledged in the Results and later in the Discussion.<br>The sample spans from early childhood to late adolescence. Despite this, the Results section reports aggregated statistics without stratification by age group or developmental stage. Because feeding behaviors, HEI scores, and parental stress vary significantly with child age, subgroup analyses or at least age-adjusted associations would markedly improve the validity of the findings. As currently presented, some results may mask or dilute age-related differences.<br><br>Lacking reporting confidence intervals, distinguishing between statistically significant vs clinically meaningful associations, and indicating the magnitude of effects (e.g., whether correlations are minor, moderate, or large). At present, the emphasis is solely on p-values, which limits interpretability. Although most causal statements occur in the Discussion, a few sentences in the Results implicitly suggest directional effects (e.g., “preparing special meals was negatively associated with HEI”), but without explicit caution that the design is cross-sectional. The results should remain strictly descriptive, reserving interpretation for the discussion.<br><br>Also, since the richness of the data, the Results would benefit from at least one figure (e.g., scatterplots of key correlations, or a bar graph of HEI categories). This is not mandatory, but it would greatly aid readability. Consider adding visual depictions of the main results.<br><br>DISCUSSION<br><br>The discussion interprets results and compares them with prior research, but could be strengthened in three areas: distinguishing between correlation and causation and avoiding sentences that imply directional effects (e.g., feeding problems predict poor diet quality), which are not appropriate for a cross-sectional design. While recommendations are mentioned, more concrete guidance (e.g., structured feeding protocols, multidisciplinary approaches, DSM-based guidelines) would increase impact.<br><br>Lack of a social validity or acceptability component. Since the study focuses on parental stress and mealtime accommodations, it would be natural and valuable to include parental acceptability, perceived usefulness, and the feasibility of feeding practices. Although data cannot be added retrospectively, the limitation should be acknowledged.<br><br>The limitations section is adequate, but could include: a lack of treatment data, a lack of response rates, and sample heterogeneity.<br><br>Other parts not clear:<br>- “Dietary Habits: Children's dietary habits were assessed, covering aspects like food allergies, taste preferences, skipping meals, and dining out frequency. It also assessed feeding difficulties such as food selectivity, refusal, chewing/swallowing issues, pica, constipation, diarrhea, food intolerance, temper outbursts, and

	hyperactivity." (How was the assessment? And scoring?)<br>- 2.2.6-8 Some scoring and item examples would increase the comprehension of the instruments in general.<br>- The sociodemographic interview is not well described.<br>- Tables in the results show differences in instruments (indirect) by children's gender. However, boys were overrepresented (have you tested for a sampling proportion difference?). Likewise, why did the age not split for the same scope?<br><br>Main issue to revise:<br>- Literature connected and updated for each domain you investigate<br>- Hypothesis<br>- Recruitment<br>- Proportion sampling test<br>- Age ranges approach<br>- Details in instruments<br>- Research design with clustering, or other models (no clear current research process model)<br>- Role of predictors<br>- Linear Sections for Results (in line with method and discussion)<br>- Current limitations, clinical implications, and future studies<br>- National and International Guidelines<br><br>In conclusion, the manuscript contributes valuable data on an understudied population and has strong potential for publication. However, several methodological clarifications, reporting improvements, and analytic refinements are necessary to ensure scientific rigor and interpretability. With substantial revision addressing the issues outlined above, the paper could become a significant contribution to the literature on feeding behavior and family well-being in ASD.<br><br>Best of Luck with your proposal

VERSION 1 – AUTHOR RESPONSE

**Reviewer 1 (yellow highlights in the manuscript)**	
Foremost, they assume the diagnosis of ASD for 111 patient but fail to document how or by whom the diagnosis was made. The reference standard is a qualified diagnostician administering an appropriate module of the Autism Diagnostic Observation Schedule, Second Edition (ADOS-2) along with standardized testing of cognitive or intelligence skills, and review of the DSM-5-TR diagnostic criteria. Withing this context, the author must then differentiate between patients which ADOS-2 substantiated ASD with average intelligence versus those who have intellectual disability (ID) (i.e., a cognitive developmental quotient (DQ) or an IQ < 70.	We appreciate this comment. We clarified in the "Participants" section that all children hold a mandatory medical board report (ÇÖZGER) issued by psychiatrists based on DSM-5 criteria, ensuring diagnostic validity. Regarding cognitive functioning, we added a statement to the Limitations section acknowledging that standardized IQ scores were not available in the rehabilitation records, preventing stratification by intellectual disability. Although this lack of data is a limitation, the fact that our sample comes from educational rehabilitation centres (special education) indicates that the participants have undergone similar support processes.
Did the patients have any comorbidities (e.g., attention-deficit/hyperactivity disorder [ADHD],	We updated Table 2 and the Results to report available comorbidity data. While formal psychiatric

generalized anxiety disorder [GAD], etc.)? The authors must address this.	diagnoses were not recorded, we now explicitly present the prevalence of epilepsy and behavioral symptoms like hyperactivity to provide a clearer clinical picture of the group.
The authors use broad, non-specific terminology of feeding problems, picky eating, and food selectivity. These are common in neurotypical children, affecting up to 40% of them. The authors must instead use feeding-related diagnoses such as pediatric feeding disorder (PFD) or, possibly, avoidant/restrictive food intake disorder (ARFID), both of which have diagnostic criteria and ICD-10 codes associated with them.	We agree that precise terminology is essential. While we retained behavioral descriptors consistent with our specific measurement tools (BAMBI), we revised the Introduction and Discussion to explicitly contextualize these findings within the PFD and ARFID frameworks, acknowledging the clinical overlap despite the absence of diagnostic interviews.
Did any of the patients have feeding-related complications (e.g., oropharyngeal dysphagia, chronic constipation, severe gastroesophagel reflux disease [GERD])? Each of these can affect a child's food intake	We updated the Results section to explicitly highlight the prevalence of physiological challenges detected in our sample, specifically reporting rates for chewing/swallowing difficulties and constipation to reflect the biological complexity of feeding issues.
Although, while the author's intention and the tone of the manuscript are commendable, the study as written lacks the scientific rigor needed to make it worthy of publication at this time.	We have substantially revised the manuscript to elevate scientific rigor. Key improvements include validating diagnostic criteria, integrating current clinical frameworks (PFD/ARFID), expanding clinical characterization with comorbidity data, and transparently discussing limitations regarding cognitive testing.
**Reviewer 2 (green highlights in the manuscript)**	
INTRODUCTION- This section lacks methodological detail in the cited studies, including sample characteristics, methodology, primary findings, and contextual relevance to the present study. This omission weakens the rationale. For example, describing sample sizes, age ranges, the types of measures used, and core results provides essential grounding and allows the reader to understand how the present work advances the field (by connecting previous studies with the current one). Furthermore, no references to the AAP Pediatric Nutrition Guidelines, CDC ASD feeding resources, or NICE ASD guidelines would enhance clinical relevance. Generally, the previous studies and a primary updated literature are recommended.<br><br>Moreover, research questions and hypotheses are not explicitly stated. The manuscript states a general aim but does not articulate specific,	We revised the Introduction to provide detailed methodological context for cited studies and integrated relevant guidelines (NICE, AAP). Furthermore, we explicitly stated three testable hypotheses (H1, H2, H3) to align the study aim with the statistical analysis.

testable hypotheses. Explicit hypotheses would improve coherence and allow more precise evaluation of the results, such as H1: Higher selective behaviors scores are associated with lower HEI scores. H2: Greater parental stress is associated with higher perceived burden. H3: Parental mealtime strategies (e.g., special meals) predict variations in diet quality. This would align well with the analyses already conducted.	
METHOD- I noted missing details on recruitment and participant flow. While the manuscript states that participants were recruited from two educational centers, several key details are missing: the number of parents approached, the number who declined, and whether consecutive sampling included all families. Any inclusion criteria regarding ASD diagnosis certification, whether children were attending behavioral, nutritional, or therapeutic interventions. These omissions limit the evaluation of selection bias. A flow diagram would be helpful, given the extensive age range and the lack of subgroup analyses. The sample ranges from 2 to 19 years, which is developmentally heterogeneous for feeding behavior research. Yet no subgroup or cluster analyses (e.g., toddlers vs. school-age vs. adolescents) were conducted, nor was a rationale provided for treating the group as homogeneous. Mainly, age strongly influences eating autonomy, sensory development, parental feeding strategies, BMI, and HEI comparability. The current research design seems weak (without comparison and models).	We clarified in the Participants section that consecutive sampling was used and noted the sample's treatment-naive status. In the Limitations, we acknowledged the lack of a recruitment log. We also addressed the age distribution, explaining that the sample predominantly represents early-to-middle childhood, which precluded valid adolescent subgroup analyses.
METHOD- The manuscript reports Cronbach's α values and cites Turkish adaptations. However, key information missing includes: item response formats (e.g., 1–5 Likert scale), interpretative cut-offs (if any), and scoring rules (sum vs. mean; handling missing items). Including this information increases transparency and reproducibility. The instruments are too synthetic, lacking examples and prior applications.	We clarified in the Participants section that consecutive sampling was used and noted the sample's treatment-naive status. In the Limitations, we acknowledged the lack of a recruitment log. We also addressed the age distribution, explaining that the sample predominantly represents early-to-middle childhood, which precluded valid adolescent subgroup analyses.
METHOD- The analytical approach is too simplistic; some points need refinement: Pearson vs Spearman correlations, HEI, and BAMBI scores often show non-normal distributions in ASD samples. If normality were	We expanded our statistical strategy to include a multivariate linear regression model, allowing us to control for potential confounders such as age and sex. We also validated normality assumptions, prioritized non-parametric tests (Mann-Whitney U) for

violated, Spearman correlations would be more appropriate. Given socioeconomic variables influenced outcomes, a model would more convincingly establish associations while controlling for confounders (income, age, BMI, sex). No reporting of effect sizes: T-tests and Mann–Whitney U tests should include: Cohen's d, r effect size, and confidence intervals. No correlation or exploratory models are applied. Also, the manuscript does not report: ASD severity, cognitive level, communication abilities, comorbidities beyond brief mention, type or intensity of therapy (ABA, sensory integration, feeding therapy). These variables are directly related to feeding behavior and stress, and their omission may lead to misinterpretation.	non-normally distributed variables, and reported effect sizes and confidence intervals to clearly demonstrate the magnitude of observed associations.
RESULTS-The Results section is generally clear and presents substantial descriptive and inferential data; however, several important issues require clarification or refinement to strengthen transparency, coherence, and interpretability. The section provides extensive raw descriptive data (e.g., anthropometrics, feeding behaviors, dietary habits, socioeconomic variables), but the narrative lacks a clear structure. The reader would benefit from clearer organization, including sections on child characteristics, feeding-related variables, parental outcomes, and the associations among variables. This would improve readability and conceptual coherence.	We reorganized the Results section into distinct subsections to improve narrative flow and conceptual coherence, ensuring the presentation clearly follows the methodological structure.
RESULTS-Moreover, the results do not include several essential variables that are typically necessary to interpret feeding behaviors in ASD, such as ASD severity levels, cognitive or adaptive functioning, the type or intensity of interventions (e.g., ABA, sensory integration, feeding therapy), communication level, and comorbidities beyond broad categories. The absence of these data limits the capacity to understand heterogeneity within the sample and may confound observed associations. At a minimum, this limitation should be acknowledged in the Results and later in the Discussion.	We explicitly stated in the Results that standardized severity scores were unavailable due to the study setting. To address this, we added a detailed paragraph to the Limitations section acknowledging how the lack of cognitive and adaptive functioning data restricts the assessment of sample heterogeneity.
RESULTS-The sample spans from early childhood to late adolescence. Despite this, the Results section reports aggregated statistics without stratification by age group or	Given the sample's skew towards early childhood, subgroup stratification was not statistically feasible. Instead, we addressed age-related variance by entering the child's age as a covariate in the

developmental stage. Because feeding behaviors, HEI scores, and parental stress vary significantly with child age, subgroup analyses or at least age-adjusted associations would markedly improve the validity of the findings. As currently presented, some results may mask or dilute age-related differences.	multivariate regression analysis, as detailed in the revised Statistical Analysis section.
RESULTS-Lacking reporting confidence intervals, distinguishing between statistically significant vs clinically meaningful associations, and indicating the magnitude of effects (e.g., whether correlations are minor, moderate, or large). At present, the emphasis is solely on p-values, which limits interpretability. Although most causal statements occur in the Discussion, a few sentences in the Results implicitly suggest directional effects (e.g., "preparing special meals was negatively associated with HEI"), but without explicit caution that the design is cross-sectional. The results should remain strictly descriptive, reserving interpretation for the discussion.	We revised the analysis to report standardized beta coefficients and 95% confidence intervals, moving beyond reliance solely on p-values. Additionally, we carefully reviewed the text to ensure all language reflects the cross-sectional design, avoiding causal assertions.
RESULTS-Also, since the richness of the data, the Results would benefit from at least one figure (e.g., scatterplots of key correlations, or a bar graph of HEI categories). This is not mandatory, but it would greatly aid readability. Consider adding visual depictions of the main results.	We revised the analysis to report standardized beta coefficients and 95% confidence intervals, moving beyond reliance solely on p-values. Additionally, we carefully reviewed the text to ensure all language reflects the cross-sectional design, avoiding causal assertions.
DISCUSSION-The discussion interprets results and compares them with prior research, but could be strengthened in three areas: distinguishing between correlation and causation and avoiding sentences that imply directional effects (e.g., feeding problems predict poor diet quality), which are not appropriate for a cross-sectional design. While recommendations are mentioned, more concrete guidance (e.g., structured feeding protocols, multidisciplinary approaches, DSM-based guidelines) would increase impact.	We carefully refined the Discussion section to distinguish between correlation and causation. We also strengthened the clinical implications by recommending structured, multidisciplinary approaches grounded in the findings.
DISCUSSION-Lack of a social validity or acceptability component. Since the study focuses on parental stress and mealtime accommodations, it would be natural and valuable to include parental acceptability, perceived usefulness, and the feasibility of feeding practices. Although data cannot be	We added a specific point to the Limitations section acknowledging the absence of social validity measures. We recommended that future research evaluate the feasibility and parental acceptability of feeding interventions.

added retrospectively, the limitation should be acknowledged.	
DISCUSSION-The limitations section is adequate, but could include: a lack of treatment data, a lack of response rates, and sample heterogeneity.	We expanded the Limitations section to explicitly acknowledge the lack of a formal recruitment log and detailed data on the intensity of concurrent non-dietary therapies, as requested.
OTHERS-- “Dietary Habits: Children's dietary habits were assessed, covering aspects like food allergies, taste preferences, skipping meals, and dining out frequency. It also assessed feeding difficulties such as food selectivity, refusal, chewing/swallowing issues, pica, constipation, diarrhea, food intolerance, temper outbursts, and hyperactivity.” (How was the assessment? And scoring?)<br>- 2.2.6-8 Some scoring and item examples would increase the comprehension of the instruments in general.<br>- The sociodemographic interview is not well described.<br>- Tables in the results show differences in instruments (indirect) by children's gender. However, boys were overrepresented (have you tested for a sampling proportion difference?). Likewise, why did the age not split for the same scope?	We clarified that clinical variables were collected via structured questionnaires and expanded instrument descriptions. Regarding gender balance, we justified the male preponderance based on epidemiological norms and used appropriate non-parametric tests to handle unequal sample sizes.
MAIN ISSUES:<br><br>- Literature connected and updated for each domain you investigate<br>- Hypothesis<br>- Recruitment<br>- Proportion sampling test<br>- Age ranges approach<br>- Details in instruments<br>- Research design with clustering, or other models (no clear current research process model)<br>- Role of predictors<br>- Linear Sections for Results (in line with method and discussion)<br>- Current limitations, clinical implications, and future studies<br>- National and International Guidelines<br><br>In conclusion, the manuscript contributes valuable data on an understudied population and has strong potential for publication. However, several methodological clarifications, reporting improvements, and analytic	**Summary of Revisions based on Main Issues:** We are grateful for the reviewer’s encouraging concluding remarks and the clear roadmap provided for revision. We have used the "Main Issues" list as a checklist to ensure every point was addressed:<br><br>• **Literature:** Updated with recent systematic reviews (Page et al., 2022; John et al., 2025) and integrated clinical definitions (PFD/ARFID).<br>• **Hypotheses:** Explicitly defined (H1, H2, H3) in the Introduction.<br>• **Recruitment:** Clarified consecutive sampling and the treatment-naive nature of the cohort.<br>• **Proportion Sampling:** Justified gender ratios via epidemiological data and utilized appropriate non-parametric tests.<br>• **Age Ranges:** Addressed the skew toward early childhood and controlled for age in the regression model.<br>• **Instruments:** Expanded details on scoring, Likert scales, and sample items.<br>• **Research Design & Predictors:** Implemented a **Multivariate Linear Regression Model** to establish the role of predictors (BAMBI) on outcomes (HEI) while controlling for confounders.

refinements are necessary to ensure scientific rigor and interpretability. With substantial revision addressing the issues outlined above, the paper could become a significant contribution to the literature on feeding behavior and family well-being in ASD.	• **Results Structure:** Reorganized into linear subsections corresponding to the methods.<br>• **Guidelines:** Integrated NICE and AAP guidelines to enhance clinical relevance.<br><br>We believe these substantial revisions have directly addressed the methodological and reporting gaps, ensuring the scientific rigor required for publication.

VERSION 2 – REVIEW

**REVIEWER NAME**	Phalen, James
**REVIEWER AFFILIATION**	
**REVIEWER CONFLICT OF INTEREST**	No Conflict of Interest
**AI DISCLOSURE**	No, I have not used AI
**DATE REVIEW RETURNED**	25-Jan-2026

**GENERAL COMMENTS**	Dear authors,<br><br>You significantly improved the quality of your manuscript based on recommendations from the other reviewer and myself. I now find that this is more substantial, readable, and that it warrants publication. Congratulations on your efforts.

**REVIEWER NAME**	Esposito, Marco
**REVIEWER AFFILIATION**	Dep. Life of Sciences
**REVIEWER CONFLICT OF INTEREST**	No Conflict of Interest
**AI DISCLOSURE**	Grammarly for no native speaker (grammatical correction)
**DATE REVIEW RETURNED**	15-Jan-2026

**GENERAL COMMENTS**	Thank you for inviting me to revise the current manuscript “Mealtime behaviors, diet quality and parental stress in children with autism spectrum disorder: a cross-sectional study from Türkiye.”<br><br>This manuscript addresses a clinically important topic, namely the interplay between mealtime behaviors, diet quality, and parental stress and quality of life in families of children with autism spectrum disorder (ASD). Feeding difficulties and nutritional imbalance are well-documented challenges in ASD, yet they are often investigated in isolation from caregiver outcomes.<br><br>However, several major issues require careful and deep revision before the manuscript can be considered for publication.<br><br>I have carefully revised the manuscript point by point.<br><br>Title: consider “a survey from” or “correlational” (since the cross nature is minimal and not treated)<br><br><br><br>Introduction

	"With approximately 1 in 36 children in the United States diagnosed with ASD as of 2020 [3]. The prevalence of ASD in Türkiye is estimated to be 0.6% [4]."<br><br>Consider equivalent measures and years of epidemiology.<br><br>"Beyond the core diagnostic criteria, the lived experience of individuals with the disorder often involves a broader array of challenges. Among the most prevalent are gastrointestinal symptoms, heightened sensory sensitivities, and marked disturbances in eating behavior [5,6]."<br><br>In this manner, it seems that the main issues of ASD symptoms are GID and feeding issues. Please enrich these statements.<br><br>"Importantly, the impact of feeding-related problems in ASD extends beyond the individual child, placing substantial emotional…" (all section)<br><br>Please consider increasing the citations and argumentations.<br><br>"problems than their peers[8],"<br><br>Check line 45.<br><br>Moreover, the same section is confused. The authors introduce the topic of interventions without details and debate about the chronicity of symptoms. Often, behavioral interventions are not effective without physical or sensory treatments, and in the presence of ID, alternative feed is essential. In my opinion, the author should rewrite the section, adding literature and argumentations, naturally, in line with their hypothesis and scope.<br><br>AIM and hypothesis<br><br>Firstly, regarding the aim, what does the current correlation study add to previous literature? No occidental sample?<br><br>Secondly,<br><br>"H3: Maladaptive parental mealtime strategies predict variations in diet quality."<br><br>I have problems with the term "maladaptive" since it is a clinical interpretation of parenting after a causal inference. I suggest "high demanding?" "authoritative", "pressure", "passive/accommodated") more educational terms (throughout the manuscript). In what direction is passive-demanding or qualitative emotional-encouraging?<br><br>"This distribution reflects the typical demographic of the rehabilitation centers, where early intervention is prioritized."<br><br>Not only prioritized but I suppose "offered" from health services than social assistance for young or adult services.<br><br>Participants<br><br>Clinical characterization of the ASD sample: the manuscript lacks key clinical descriptors that are highly relevant to feeding behavior

	and diet quality in ASD. Specifically, no information is provided regarding: ASD severity or level of support needs, presence or absence of intellectual disability, functional language level, adaptive functioning, or intensity and type of ongoing interventions (e.g., behavioral therapy, occupational therapy, speech therapy). These variables are well known to influence feeding selectivity, mealtime behavior, caregiver stress, and nutritional outcomes. Their absence limits both internal validity (risk of unmeasured confounding) and external validity (comparability with other ASD samples). If available, include at least proxy indicators (e.g., DSM-5 support level, verbal vs minimally verbal status). If such data cannot be retrieved, this limitation should be emphasized more explicitly and earlier (Methods and Discussion), rather than primarily in the concluding limitations section. Clarify that findings apply to a clinically heterogeneous “real-world” rehabilitation sample rather than a phenotypically stratified ASD cohort.<br><br>Was data collected online or on the web platform?<br><br>HEI-2020<br><br>Dietary assessment and use of HEI-2020: the use of the Healthy Eating Index–2020 is an important strength, but the manuscript currently provides insufficient methodological detail regarding how HEI scores were derived in this cultural context. For example, the nutritional database used to code food records, how Turkish foods and mixed dishes were mapped onto HEI-2020 components, whether portion sizes were estimated using standardized household measures, who performed dietary coding, and whether inter-rater reliability was assessed. Given that HEI-2020 is based on U.S. dietary guidelines, its application in a Turkish pediatric ASD sample requires explicit justification and a transparent description. Please, add a short subsection in Methods detailing dietary coding procedures and quality control. Likewise, briefly discuss the appropriateness and limitations of HEI-2020 in non-U.S. contexts, citing any international applications where possible. How were the responses of parents? Finally, clarify whether HEI was used as an absolute measure of diet quality or as a comparative index within the sample.<br><br>“Meral and Fidan (2014)[26].”<br><br>Check the line.<br><br>“The internal consistency of the Turkish form was found to be satisfactory, with a Cronbach’s alpha coefficient of .79, and split-half reliability coefficients were calculated as .86 and .83.”<br><br>Please, the authors should include the current reliability (as for other questionnaires).<br><br>“(2009)[27],”<br><br>Check the line.<br><br>“Reliability analysis for the Turkish version showed Cronbach’s alpha coefficients for the sub-dimensions ranging between .41 and .75.”<br><br>Idem, as afore mentioned.

	“from 'Never' (1) to 'Always'.”<br><br>Check the line<br><br>“The psychometric analysis demonstrated high internal consistency, yielding a Cronbach’s alpha value of .85 and a Spearman-Brown split-half reliability of .82.”<br><br>In this case, I suggest checking also the factorial dimensions since you could utilize more dimensions in favor of your test and interpretation of hypotheses.<br><br>“Quality of Life in Autism Questionnaire (QoLA) ?”<br><br>I suggest better explaining the Part B data collection.<br><br><br>Data analysis<br><br>“Participants with substantially incomplete questionnaires were excluded from the study prior to analysis.”<br><br>How many participants?<br><br>Moreover, the variables selected (type, levels, hypothesis position, and inclusion in the tests) are not described.<br><br>“All data were analyzed using SPSS version 22.0. Participants with substantially incomplete questionnaires were excluded from the study prior to analysis. … Correlations between continuous variables were assessed using Pearson’s correlation coefficient (r). A p-value of less than 0.05 was considered statistically significant.”<br><br>This section should be rewritten as afore suggested, in line with the aim and hypotheses.<br><br>“of the precision of the estimated relationships.”<br><br>Check the line.<br><br>After having rewritten the first section, the other two should be aligned with the prior.<br><br>Statistical modeling and alignment with hypotheses: the manuscript presents three hypotheses (H1–H3), but the analytic strategy does not consistently or explicitly test each hypothesis with a corresponding statistical model. Additionally, H3 proposes that maladaptive parental mealtime strategies predict diet quality. While correlations between PMAS subscales and HEI components are presented, PMAS variables are not included in the multivariate regression model predicting HEI. Either include PMAS subscales (e.g., “Special Meals”, “Snack Provision”) in an adjusted regression or reframe H3 and its conclusions in correlational rather than predictive terms. Finally, hypothesis H2 (parental stress and burden), associations between PSS and QoLA-B are described, but it is unclear whether these were tested in a multivariate framework controlling for child characteristics. Clarify whether H2 was evaluated via regression or correlation only, and align interpretation

	accordingly.<br><br>RESULTS<br><br>"Evaluation of demographic characteristics of children and families"<br><br>One point, Why the sample of children(by age) were not split in preschool and primary school? Perhaps there were not enough participants?<br><br>"According to BMI classification, 12.6% were underweight, 45.9% had a normal weight, and 41.4% were overweight or obese."<br><br>BMI results should report even Z scores.<br><br>"Notably, 70.3% of children were reported as picky eaters, and 29.7% were reported to skip main meals."<br><br>It is necessary to provide a context regarding the dichotomous questions.<br><br>"The most common medical comorbidity was epilepsy (6.3%). While formal psychiatric diagnoses such as Attention-Deficit/Hyperactivity Disorder (ADHD) or Generalized Anxiety Disorder (GAD) were not reported in the medical history, behavioral manifestations associated with these conditions were prevalent; 56.8% of the children exhibited hyperactivity and 27.0% displayed temper tantrums (Table 2)."<br><br>Although at the correlational level (survey), I note that comorbidities could be included as cofounders, could they not?<br><br>"Table 2. Assessment of health information and dietary habits of children and families"<br><br>Why do the authors compare by sex rather than by age or age of parents? In such a sense, the sample could be more representative.<br><br>Gender comparisons and interpretative caution: The finding that parents of girls with ASD report higher stress and burden is intriguing. However, the gender comparison is based on a markedly unbalanced sample (85 boys vs 26 girls), with significant age differences between groups. Without adjustment for age, socioeconomic variables, or ASD severity, these findings should be interpreted cautiously.<br><br>Have you tried to split the entire sample by BAMBI median? PMAS and QoL scores could be more clinically interesting than by sex. Likewise, I note a discrepancy between hypotheses and tables (you could include the current table by sex in the appendix and leave merely a description (sociodemographic in the text).<br><br>H1: Higher scores in maladaptive mealtime behaviors are associated with lower diet quality.<br><br>H2: Greater parental stress is positively associated with a higher perceived autism-related burden.<br><br>H3: Maladaptive parental mealtime strategies predict variations in diet quality

	Since my suggestions are radical, driving the analysis toward a more linear clinical model, you should consult with the editor before revising the paper.<br><br>Discussion<br><br>First, the Discussion places substantial interpretative weight on the multivariate regression model predicting diet quality (HEI-2020), despite the relatively modest proportion of explained variance. While a low-to-moderate R^2 is not uncommon in cross-sectional psychosocial research, particularly in heterogeneous neurodevelopmental populations, the current framing risks overstating the explanatory value of the model.<br><br>Diet quality in children with ASD is widely recognized as a multifactorial outcome shaped by neurodevelopmental severity, sensory sensitivities, gastrointestinal comorbidities, functional communication, adaptive skills, family routines, and access to interventions. Several recent reviews emphasize that feeding-related outcomes in ASD cannot be meaningfully interpreted without situating behavioral predictors within this broader clinical and contextual framework. In this light, the regression findings should be discussed more explicitly as exploratory and associative, rather than as evidence of robust predictive pathways.<br><br>Relatedly, the Discussion occasionally adopts language that implies directional or causal mechanisms (e.g., reinforcement cycles, parental strategies perpetuating poor diet quality). While such interpretations are theoretically plausible and well-grounded in behavioral and family-systems models, they are not directly testable within the present cross-sectional design. A more cautious framing emphasizing bi- directionality and transactional processes between child behavior and parental responses would better align the Discussion with both the study design and contemporary conceptual models highlighted in recent narrative and systematic reviews.<br><br>The interpretation of parental mealtime strategies warrants particular attention. The Discussion tends to characterize certain strategies as maladaptive, implicitly attributing a negative functional role to parental behaviors. However, this terminology risks pathologizing parental responses without sufficient empirical support, especially given the absence of longitudinal or experimental data. Recent reviews on feeding interventions in ASD underscore that many parental accommodations emerge as adaptive short-term strategies in response to severe feeding refusal, sensory sensitivities, or medical concerns. Reframing these strategies as context-dependent responses rather than inherently maladaptive behaviors would add conceptual nuance and reduce the risk of causal oversimplification.<br><br>The discussion of parental stress and quality of life is one of the strongest elements of the manuscript, yet it could be further strengthened by a clearer theoretical positioning. The findings suggest that parental stress is more closely linked to perceived burden and quality-of-life impairment than to isolated child feeding behaviors per se. This aligns with transdiagnostic and family-

	centered models described in recent reviews, which conceptualize caregiver stress as the product of cumulative demands rather than single child-level symptoms. Making this link more explicit would elevate the Discussion from a descriptive comparison to a theoretically informed interpretation.<br><br>Gender-related findings are discussed with interest, but the current narrative risks overinterpretation. Given the unbalanced sample size and age differences between boys and girls, the Discussion should consistently frame these results as exploratory and hypothesis-generating. Recent literature on gender differences in ASD emphasizes diagnostic and referral biases, as well as differential parental expectations, which may influence caregiver-reported stress. Integrating this perspective would provide a more balanced and literature-consistent interpretation.<br><br>The use of HEI-2020 as a global diet quality indicator is appropriately highlighted as a strength, but the Discussion would benefit from a more explicit acknowledgment of its limitations in non-U.S. contexts. Recent reviews on nutritional assessment in ASD note that while composite indices are useful for comparative purposes, they may not fully capture culturally specific dietary patterns or clinically relevant nutrient inadequacies. Positioning HEI-2020 as a comparative, rather than normative, indicator within this sample would enhance methodological transparency and strengthen the credibility of the conclusions drawn.<br><br>Finally, the Discussion would be strengthened by a more explicit integration of recent systematic reviews published in the last five years on feeding disorders, ARFID, and diet quality in neurodevelopmental conditions. While several key studies are cited, the absence of a stronger review-based synthesis limits the interpretative depth of the section. Incorporating these reviews would not only contextualize the findings more robustly but also clarify how the present study advances the field beyond existing evidence.<br><br><br>Minor suggestions<br><br>Abstract: After revision, consider rewriting the abstract with statistical and design adjustments, specifying the role of HEI from 3-day food records.<br><br>Introduction: Please include at least 5 recent reviews on ARFID/feeding disorder in neurodevelopmental conditions, multilevel interventions and behavioral on feeding problems in ASD, familial and stress outcomes associated with feeding problems. Also, the authors may include a review on diet quality in ASD to justify the utilization of HEI as the main outcome (diet quality in ASD, nutrient adequacy vs. patterns).<br><br>Concluding<br><br>The manuscript furnishes data from a survey (not well specified in the text) in two clinical samples of ASD (preschool and primary). Data are collected by reliable measures, although self-reports. The introduction is lacking in literature. The hypotheses do not sufficiently guide the manuscript development. The survey data

	(table by sex) should be revised, explaining why the sample was split by sex (and why by sex only). Why were comorbidities not included in the model? Moreover, a deep revision of the data analysis section. Could the manuscript be reorganized moved several pieces of information, and the tables in the appendix or supplemental material?<br><br>In my opinion, your proposal is publishable after major revisions, but you should take efforts to align hypothesis-research design-analysis.<br><br>The main additional motive is the non-occidental sample and a more idiosyncratic mealtime context, but the availability of data could be more clinically guided.<br><br>I hope my suggestion will improve the quality of your proposal.<br><br>Good Luck

VERSION 2 – AUTHOR RESPONSE

Reviewer 2:

-Consider "a survey from" or "correlational" (since the cross nature is minimal and not treated):

We sincerely thank the reviewer for this thoughtful methodological observation. We agree that our primary analytical approach focuses on the interrelationships and correlations among variables rather than comparing distinct cross-sections of the population.

However, we respectfully prefer to retain the term "Cross-Sectional Study" in the title for several fundamental methodological reasons. In epidemiological and clinical research, the term "cross-sectional" primarily defines the timing of data collection—specifically, that all data were gathered at a single point in time without any longitudinal follow-up. Furthermore, our study was strictly designed, conducted, and reported in accordance with the STROBE guidelines specifically for cross-sectional studies. Changing the title to correlational or survey might disconnect our manuscript from this standardized epidemiological reporting framework. Additionally, maintaining cross-sectional in the title transparently alerts readers upfront to the study's inherent limitations regarding causal inferences, which we discuss in the limitations section.

To accommodate your valuable feedback, we have ensured that the relational and correlational nature of our analyses is explicitly emphasized in the Methods and Abstract sections, clarifying that while the structural design is cross-sectional, the analytical framework is predominantly correlational. We hope the reviewer agrees with this methodological justification.

-"With approximately 1 in 36 children in the United States diagnosed with ASD as of 2020 [3]. The prevalence of ASD in Türkiye is estimated to be 0.6% [4]." Consider equivalent measures and years of epidemiology.

We have revised the introduction to ensure consistency between the international and national prevalence data.

- "Beyond the core diagnostic criteria, the lived experience of individuals with the disorder often involves a broader array of challenges. Among the most prevalent are gastrointestinal symptoms, heightened sensory sensitivities, and marked disturbances in eating behavior [5,6]." In this manner, it seems that the main issues of ASD symptoms are GID and feeding issues. Please enrich these statements.

We agree with the reviewer that the distinction between core diagnostic criteria and co-occurring conditions should be more explicit. We have revised the text to clearly define the core symptoms of ASD while framing gastrointestinal and feeding issues as prevalent comorbid factors that contribute to the clinical heterogeneity and overall burden of the disorder.

Revised text (Introduction):

Beyond the core diagnostic criteria of social communication deficits and restricted, repetitive behaviors, ASD is characterized by a complex clinical heterogeneity that frequently involves various co-occurring conditions. Among these, non-core symptoms such as gastrointestinal disturbances, heightened sensory sensitivities, and marked disturbances in eating behavior are highly prevalent and significantly contribute to the overall disease burden [5,6]. While not part of the primary diagnostic markers, these physiological and behavioral challenges often interact with core ASD symptoms, creating a multifaceted impact on both the individual's daily functioning and the family's quality of life.

-Importantly, the impact of feeding-related problems in ASD extends beyond the individual child, placing substantial emotional…" (all section) . Please consider increasing the citations and argumentations.

We have extensively expanded the section discussing the familial and emotional impact of feeding problems. By incorporating evidence from Gent et al. (2023) and Guller & Yaylaci (2022), we now argue that feeding challenges are among the strongest predictors of caregiver daily life disruption and psychological distress, often exceeding the impact of core autism symptoms. The revised section provides a more nuanced look at how chronic mealtime stress influences family resilience and parental coping strategies, as supported by Amate & Rosa (2024)

-"problems than their peers[8]," Check line 45. Moreover, the same section is confused. The authors introduce the topic of interventions without details and debate about the chronicity of symptoms. Often, behavioral interventions are not effective without physical or sensory treatments, and in the presence of ID, alternative feed is essential. In my opinion, the author should rewrite the section, adding literature and argumentations, naturally, in line with their hypothesis and scope.

We have added detailed argumentation on why behavioral interventions must be integrated with sensory and physical treatments, citing the central role of atypical sensory responsivity in feeding difficulties (Consoli et al., 2025). Additionally, we now discuss the necessity of multidisciplinary, multicomponent strategies (Chung et al., 2024) and specifically address the clinical requirement for alternative feeding in the presence of intellectual disability or severe ARFID presentations, providing a more comprehensive clinical perspective as requested.

Firstly, regarding the aim, what does the current correlation study add to previous literature? No occidental sample?

We thank the reviewer for these insightful comments. We have revised the manuscript as follows:

-The Introduction now emphasizes our focus on how specific levels of feeding disturbances selectively relate to diet quality and parental burden, moving beyond general associations to provide a more granular analysis.

-We have rewritten the section on ASD-related comorbidities to clearly distinguish core symptoms from co-occurring feeding and GI issues.

Revised text (Introduction):

The primary objective of this study is to examine the associations between feeding behaviors in children with ASD, their dietary quality, and the subsequent psychosocial burden on their caregivers (H1, H2, and H3). While the co-occurrence of these challenges is recognized, the present study seeks to provide a more nuanced evaluation by identifying how specific levels of mealtime behavioral disturbances relate to various domains of parental quality of life and children's nutritional status. By investigating these relationships, this study aims to contribute to the existing literature through a detailed analysis of the interplay between child-centered behavioral symptoms and parent-centered stress factors. The findings are expected to offer a clearer understanding of the multifaceted impact of feeding difficulties, potentially identifying critical areas where parental perceived burden and children's diet quality are most affected.

Revised text (Discussion):

Specifically, our results demonstrate that higher levels of mealtime behavioral disturbances are directly associated with a significant decline in children's diet quality, as measured by the HEI-2020. To further refine these observations and address the clinical heterogeneity within the sample, we examined these relationships by stratifying participants based on the median BAMBI score. This analysis revealed that the impact of feeding challenges is not uniformly distributed across all domains of life quality. While child diet quality and autism-related parental burden showed significant sensitivity to increased behavioral severity, general parental wellbeing remained relatively stable. These results suggest that the psychosocial toll of feeding difficulties may be context-specific, primarily targeting the perceived burden of autism-related care rather than broader social or economic aspects of life quality.

-H3: Maladaptive parental mealtime strategies predict variations in diet quality." I have problems with the term "maladaptive" since it is a clinical interpretation of parenting after a causal inference. I suggest "high demanding?" "authoritative", "pressure", "passive/accommodated") more educational terms (throughout the manuscript). In what direction is passive-demanding or qualitative emotional-encouraging?

We completely agree with the reviewer's perspective that "maladaptive" carries an unintended clinical judgment. We have replaced this term throughout the manuscript with more descriptive, educational labels.

Revised hypothesis:

H3: Controlling or accommodative parental mealtime strategies predict variations in children's diet quality.

-This distribution reflects the typical demographic of the rehabilitation centers, where early intervention is prioritized." Not only prioritized but I suppose "offered" from health services than social assistance for young or adult services.

We agree that the predominance of younger children in our sample is not merely a matter of prioritization by the centers, but a reflection of the systemic structure where intensive support is primarily offered as a health-funded service for early childhood. We have revised the text to better reflect this structural nuance.

Revised Text (Methods, Section 2.1):

This distribution reflects the typical demographic of the special education and rehabilitation centers in Türkiye. In this context, intensive support is primarily offered and structured for early childhood through health-funded services, whereas social assistance and structured programs for adolescents or adults remain relatively more limited.

-Clinical characterization of the ASD sample: the manuscript lacks key clinical descriptors that are highly relevant to feeding behavior and diet quality in ASD. Specifically, no information is provided regarding: ASD severity or level of support needs, presence or absence of intellectual disability, functional language level, adaptive functioning, or intensity and type of ongoing interventions (e.g., behavioral therapy, occupational therapy, speech therapy). These variables are well known to influence feeding selectivity, mealtime behavior, caregiver stress, and nutritional outcomes. Their absence limits both internal validity (risk of unmeasured confounding) and external validity (comparability with other ASD samples). If available, include at least proxy indicators (e.g., DSM-5 support level, verbal vs minimally verbal status). If such data cannot be retrieved, this limitation should be emphasized more explicitly and earlier (Methods and Discussion), rather than primarily in the concluding limitations section. Clarify that findings apply to a clinically heterogeneous "real-world" rehabilitation sample rather than a phenotypically stratified ASD cohort.

We completely agree with the reviewer that clinical descriptors such as ASD severity, intellectual disability status, and functional language are critical variables influencing feeding behavior and parental stress. Unfortunately, these specific standardized scores were not recorded in the available rehabilitation center files at the time of data collection. Following your valuable suggestion, we have now explicitly stated this limitation in the Methods section (Section 2.1) and the Discussion section. We have also clarified that our sample represents a heterogeneous "real-world" clinical population, which, while limiting phenotypic stratification, provides insight into the diverse challenges faced in typical rehabilitation settings in Türkiye.

Revised text (Methods 2.1.):

While all children in the study had a formally verified diagnosis of ASD as per national regulations, specific clinical phenotyping such as standardized ASD severity scores, cognitive functioning, functional language levels, or adaptive functioning scores were not available in the rehabilitation center records during the study period. Consequently, our findings should be interpreted as applying to a clinically

heterogeneous, real-world rehabilitation sample rather than a phenotypically stratified ASD cohort. This lack of phenotypic stratification is a recognized limitation that may introduce unmeasured confounding variables.

Revised text (Discussion,Limitations):

Second, although all participants had a clinically verified ASD diagnosis, the absence of data on ASD severity levels and intellectual disability limits our ability to identify whether specific feeding phenotypes are more prevalent in certain ASD subgroups. Future research should utilize phenotypically stratified cohorts to better understand the nuances of diet quality across the autism spectrum.

-Was data collected online or on the web platform?

We appreciate the reviewer's query regarding the mode of data collection. As stated in the Methods section (Section 2.2), all data were collected through structured, face-to-face interviews. Specifically, the manuscript mentions: *"Data were collected through structured, face-to-face interviews conducted with parents by trained researchers"*. Therefore, no online or web-based platforms were utilized for the data collection process.

-Currently provides insufficient methodological detail regarding how HEI scores were derived in this cultural context. For example, the nutritional database used to code food records, how Turkish foods and mixed dishes were mapped onto HEI-2020 components, whether portion sizes were estimated using standardized household measures, who performed dietary coding, and whether inter-rater reliability was assessed. Given that HEI-2020 is based on U.S. dietary guidelines, its application in a Turkish pediatric ASD sample requires explicit justification and a transparent description. Please, add a short subsection in Methods detailing dietary coding procedures and quality control. Likewise, briefly discuss the appropriateness and limitations of HEI-2020 in non-U.S. contexts, citing any international applications where possible. How were the responses of parents? Finally, clarify whether HEI was used as an absolute measure of diet quality or as a comparative index within the sample.

We have enriched the HEI-2020 section in the Methods to provide more granular detail on dietary coding. We utilized the BeBiS software, a comprehensive nutritional database for Türkiye, to analyze the food records. To ensure cultural alignment with the HEI-2020 components, we leveraged the Turkey Dietary Guidelines (TÜBER) for standardized portion sizes and decomposed traditional Turkish mixed dishes into their constituent ingredients. These procedures, conducted by trained dietitians, ensured that the HEI-2020 served as a robust comparative index for our sample.

Revised text (Methods 2.2.2.):

Dietary data from 3-day food records were analyzed using the Computer-Aided Nutrition Program (BeBiS), which is specifically adapted for the Turkish population and includes local recipes. To ensure accuracy in mapping Turkish mixed dishes to HEI-2020 components, recipes were decomposed into their primary ingredients. Portion sizes were estimated using standardized household measures and visual aids from the Turkey Dietary Guidelines (TÜBER) to minimize reporting bias. Dietary coding was performed by trained dietitians, and HEI-2020 was utilized as a comparative index to evaluate the relationship between mealtime behaviors and diet quality within this specific clinical sample.

-Meral and Fidan (2014)[26].” Check the line.

We corrected the spelling errors.

-The internal consistency of the Turkish form was found to be satisfactory, with a Cronbach’s alpha coefficient of .79, and split-half reliability coefficients were calculated as .86 and .83.” Please, the authors should include the current reliability (as for other questionnaires).

Thank you for this valuable methodological recommendation. We completely agree that reporting the internal consistency based on our specific sample is essential for demonstrating the reliability of our data.

Accordingly, we have calculated the Cronbach’s alpha coefficients for all psychometric scales using our current sample data (n=111). The analyses yielded acceptable to excellent internal consistency across the instruments:

- Brief Autism Mealtime Behavior Inventory (BAMBI): α = 0.651
- Parent Mealtime Action Scale (PMAS): α = 0.617
- Parental Stress Scale (PSS): α = 0.821
- Quality of Life in Autism (QoLA) Part A: α = 0.922
- Quality of Life in Autism (QoLA) Part B: α = 0.832

We have now added these sample-specific reliability coefficients to the end of each relevant subsection within the "2.2. Data Collection Instruments" section of the revised manuscript

-2009)[27],”Check the line.

We corrected the spelling errors.

“-rom 'Never' (1) to 'Always'.” Check the line

We corrected the spelling errors.

-The psychometric analysis demonstrated high internal consistency, yielding a Cronbach’s alpha value of .85 and a Spearman-Brown split-half reliability of .82.” In this case, I suggest checking also the factorial dimensions since you could utilize more dimensions in favor of your test and interpretation of hypotheses.

We thank the reviewer for this suggestion. However, the validated Turkish version of the Parental Stress Scale (PSS) by Kaymak Özmen and Özmen (2012) is specifically structured and scored as a unidimensional instrument.

-Quality of Life in Autism Questionnaire (QoLA) ? I suggest better explaining the Part B data collection.

We have rewritten the description of the QoLA questionnaire in the Methods section (Section 2.2.7). Redundant sentences were removed, and we have clearly explained that Part B items are reverse-scored in terms of burden (i.e., a score of 5 indicates "not a problem at all," meaning higher scores reflect fewer problems and a lower perceived burden).

-Participants with substantially incomplete questionnaires were excluded from the study prior to analysis. How many participants?

We thank the reviewer for this question. Since data were collected via face-to-face interviews, any substantially incomplete questionnaires were discarded immediately during the data collection phase and were not entered into the database. Therefore, we do not have an exact record of the excluded number. Data collection simply continued until our target sample size of fully completed questionnaires was reached. To avoid any confusion, we have removed the sentence regarding exclusion and clarified the text in the Methods section to simply state: *"Data collection yielded a total of 111 fully completed and valid questionnaires, which constituted the final sample for the analyses."*

-Moreover, the variables selected (type, levels, hypothesis position, and inclusion in the tests) are not described.

We agree that a more explicit description of the variables and their roles within the analytic framework was needed. We have revised the Statistical Analysis section (Section 2.3) to clearly define the outcome variables (HEI-2020 and PSS), the predictor variables (BAMBI and PMAS), and the covariates (age, BMI percentile, and income). We have also aligned these variables with their respective positions in testing H1, H2, and H3 to ensure a transparent connection between our hypotheses and the statistical tests performed.

Revised text (Methods, 2.3.):

The analytic strategy was structured to test the study's three primary hypotheses:

1. To address H1 and H2, bivariate associations between children's mealtime behaviors (BAMBI), parental mealtime strategies (PMAS), parental stress (PSS), and quality of life (QoLA-B) were assessed using Pearson's correlation coefficient.
2. To test H3, a multivariate linear regression analysis was conducted to identify independent predictors of diet quality (HEI-2020). In this model, BAMBI scores and PMAS subscales (e.g., 'Special Meals') were entered as independent (predictor) variables, while child characteristics (age, BMI-z score) and household income were included as covariates (control variables) to account for potential confounding.
3. Group comparisons (e.g., gender-based differences) were performed using the independent samples t-test for normally distributed variables or the Mann-Whitney U test for non-normal distributions.

-All data were analyzed using SPSS version 22.0. Participants with substantially incomplete questionnaires were excluded from the study prior to analysis. … Correlations between continuous variables were assessed using Pearson's correlation coefficient (r). A p-value of less

than 0.05 was considered statistically significant." This section should be rewritten as afore suggested, in line with the aim and hypotheses.

We have completely restructured the Statistical Analysis section (Section 2.3). The revised text now explicitly links each statistical method to the corresponding research hypothesis (H1, H2, and H3). It clearly defines the roles of all variables within the multivariate framework (independent predictors like BAMBI and PMAS, dependent outcome like HEI-2020, and covariates like age, and income), providing the requested methodological transparency.

-"of the precision of the estimated relationships." Check the line.

We appreciate the reviewer's attention to this detail. We have completely revised the data visualization paragraph at the end of the Statistical Analysis section. Since we updated our visualization strategy from scatter plots to median-split boxplots (grouping participants into "Low BAMBI" and "High BAMBI" based on the median score), the previous confusing terminology has been entirely removed. The revised text now accurately describes the generation of boxplots to visually compare these clinical groups across key study variables.

After having rewritten the first section, the other two should be aligned with the prior.

We have completely restructured the Statistical Analysis section (Section 2.3). Instead of a generic list of tests, the revised text now explicitly links each statistical method to the corresponding research hypothesis (H1, H2, and H3). This revision also clearly defines the roles of all variables (independent, dependent, and covariates) within the multivariate framework, providing the methodological transparency requested.

Statistical modeling and alignment with hypotheses: the manuscript presents three hypotheses (H1–H3), but the analytic strategy does not consistently or explicitly test each hypothesis with a corresponding statistical model. Additionally, H3 proposes that maladaptive parental mealtime strategies predict diet quality. While correlations between PMAS subscales and HEI components are presented, PMAS variables are not included in the multivariate regression model predicting HEI. Either include PMAS subscales (e.g., "Special Meals", "Snack Provision") in an adjusted regression or reframe H3 and its conclusions in correlational rather than predictive terms. Finally, hypothesis H2 (parental stress and burden), associations between PSS and QoLA-B are described, but it is unclear whether these were tested in a multivariate framework controlling for child characteristics. Clarify whether H2 was evaluated via regression or correlation only, and align interpretation accordingly.

We appreciate the reviewer's methodological rigor and the suggestion to better align our hypotheses with the statistical models. Following your guidance, we have updated our multivariate regression analysis (Table 5) to include Parental Mealtime Strategies (PMAS) as an independent predictor of diet quality alongside child behaviors (BAMBI). This expanded model confirmed our third hypothesis, demonstrating that PMAS independently predicts variations in diet quality even when controlling for

child-level factors and sociodemographic covariates. To ensure the reliability of this broader model, we performed comprehensive diagnostic tests, confirming that the model meets all assumptions for normality, homoscedasticity, and independence, with exceptionally low multicollinearity.

Regarding H2, we clarify that the association between parental stress (PSS) and perceived autism-related burden (QoLA-B) was evaluated solely via bivariate Pearson's correlation, which revealed a highly significant relationship ($r = -0.424$, $p < 0.001$). We have updated the Results section (Section 3.4) to explicitly reflect this finding.

Revised text(Methods, Statistics):

Following the correlation analysis, a multivariate linear regression analysis was conducted to identify the independent predictors of diet quality (HEI-2020). The model included children's mealtime behaviors (BAMBI) and parental mealtime strategies (PMAS) as the primary independent variables, while adjusting for key sociodemographic covariates: child's age, BMI-Z score, and household income. To ensure model stability and prevent overfitting, other psychosocial variables (QoLA-B) were evaluated via bivariate correlations. The validity of the regression model was rigorously verified against fundamental assumptions; multicollinearity was ruled out as Variance Inflation Factor (VIF) values for all predictors were below 1.25. The normality of residuals was confirmed by the Shapiro-Wilk test ($p=0.353$), and homoscedasticity was verified through the Breusch-Pagan test ($p=0.393$). Results were evaluated using R^2, standardized regression coefficients (β), and significance levels ($p<0.05$).

-Evaluation of demographic characteristics of children and families" One point, Why the sample of children(by age) were not split in preschool and primary school? Perhaps there were not enough participants?

We thank the reviewer for this methodological suggestion. While we acknowledge that developmental stages (preschool vs. primary school) can influence feeding behaviors and parental stress, we decided to analyze the sample as a whole to maintain sufficient statistical power for our multivariate regression models. Splitting the sample into subgroups would have significantly reduced the number of participants per category, particularly when considering the gender imbalance (85 boys vs. 26 girls), making it difficult to achieve stable and reliable estimates in the regression analysis. However, we have now included the child's age as a continuous covariate in our multivariate models to account for these developmental differences without losing statistical power.

"According to BMI classification, 12.6% were underweight, 45.9% had a normal weight, and 41.4% were overweight or obese." BMI results should report even Z scores.

Detailed anthropometric Z-scores by gender and for the total sample are provided in Table 2.

"Notably, 70.3% of children were reported as picky eaters, and 29.7% were reported to skip main meals." It is necessary to provide a context regarding the dichotomous questions.

We agree that providing clinical context for these dichotomous items is essential for accurate interpretation. We have revised the Methods section (Section 2.2.1) to clarify that these items were explained to parents using standardized descriptions of picky eating and meal skipping behaviors during the face-to-face interviews. These questions were intended to capture the parents' overall perception of their child's dietary habits, providing a descriptive baseline for the sample's clinical characterization.

Revised Text (Methods 2.2.1)

The assessment of specific dietary behaviors, such as picky eating and meal skipping, was based on parental reports using standardized definitions provided during the interview. Picky eating was defined as the consistent consumption of a limited variety of foods or the rejection of familiar/unfamiliar foods, while meal skipping referred to the child regularly missing at least one of the three primary daily meals (breakfast, lunch, or dinner). These dichotomous questions were used to provide a clinical characterization of the sample's eating patterns rather than as diagnostic tools for feeding disorders.

“The most common medical comorbidity was epilepsy (6.3%). While formal psychiatric diagnoses such as Attention-Deficit/Hyperactivity Disorder (ADHD) or Generalized Anxiety Disorder (GAD) were not reported in the medical history, behavioral manifestations associated with these conditions were prevalent; 56.8% of the children exhibited hyperactivity and 27.0% displayed temper tantrums (Table 2).” Although at the correlational level (survey), I note that comorbidities could be included as cofounders, could they not?

We appreciate the reviewer's suggestion to include comorbidities as confounders. However, as noted in the results, medical comorbidities such as epilepsy were present in only 6.3% of the sample, representing a highly unbalanced distribution. From a statistical standpoint, including variables with such low prevalence and high sparsity regression model can lead to unstable coefficients and overfitted models. Similarly, behavioral manifestations like hyperactivity were so prevalent (56.8%) that they functioned more as a core clinical characteristic of the sample rather than an independent confounding variable. Thus, we prioritized model parsimony and stability by including only sociodemographic covariates with sufficient variance.

“Table 2. Assessment of health information and dietary habits of children and families” Why do the authors compare by sex rather than by age or age of parents? In such a sense, the sample could be more representative.

We appreciate this constructive comment. Our decision to present comparisons by sex was driven by two primary factors:

-Research in ASD frequently highlights sexual dimorphism in clinical presentation, sensory profiles, and parental stress levels. Given the significant findings in our sample regarding higher stress in parents of girls, we believed sex-based comparisons provided more immediate clinical insight.

- Instead of splitting the table by age groups, which would have further fragmented the sample size and reduced statistical power for each group, we addressed age as a continuous variable in our multivariate regression models. This allowed us to control for the confounding effect of age more precisely than a categorical split would permit. However, we acknowledge the reviewer's point on representativeness. While we have kept the sex-based comparison in Table 2 to align with our specific findings on parental stress, we have ensured that the descriptive statistics for age and other demographic factors for the total sample are clearly highlighted to maintain overall representativeness

Gender comparisons and interpretative caution: The finding that parents of girls with ASD report higher stress and burden is intriguing. However, the gender comparison is based on a markedly unbalanced sample (85 boys vs 26 girls), with significant age differences between groups. Without adjustment for age, socioeconomic variables, or ASD severity, these findings should be interpreted cautiously.

We thank the reviewer for this critical and valid point. We acknowledge that the unbalanced sample size (85 boys vs. 26 girls) and the age discrepancy between groups are significant limitations that could influence the observed gender differences in parental stress. Following your suggestion, we have revised the Discussion to adopt a more cautious tone, explicitly framing these findings as exploratory. We have also highlighted that without adjustments for ASD severity or specific socioeconomic factors, these results should not be viewed as evidence of a robust causal pathway related to child gender.

Revised text (Discussion):

Our finding that parents of girls with ASD reported significantly higher stress and perceived burden (QoLA-B) compared to parents of boys is noteworthy. However, these results must be interpreted with caution due to the markedly unbalanced gender distribution in our sample (85 boys vs. 26 girls). Furthermore, significant age differences observed between the groups suggest that developmental stages may confound these gender-related perceptions. While previous literature supports potential sex-specific differences in caregiver experiences [46], our findings should be considered exploratory and hypothesis-generating rather than definitive, as they may be influenced by unmeasured variables such as ASD severity or specific socioeconomic pressures.

Have you tried to split the entire sample by BAMBI median? PMAS and QoL scores could be more clinically interesting than by sex. Likewise, I note a discrepancy between hypotheses and tables (you could include the current table by sex in the appendix and leave merely a description (sociodemographic in the text).

We sincerely thank the reviewer for this insightful suggestion. We agree that clinical severity provides a much more meaningful perspective for clinical practice than gender-based stratification. Following this recommendation, we have completely revised Figure 1. We stratified the sample into "Low BAMBI" and "High BAMBI" groups based on the median score of 42. This new approach revealed significant clinical differences across nearly all parameters (diet quality, parental stress, and mealtime strategies). Notably, while general quality of life (QoLA-Part A) did not show a significant difference, the autism-specific burden (QoLA-Part B) was significantly higher in the High BAMBI group. We believe this clinical phenotyping significantly strengthens the clinical implications of our study.

H1: Higher scores in maladaptive mealtime behaviors are associated with lower diet quality.

H2: Greater parental stress is positively associated with a higher perceived autism-related burden.

H3: Maladaptive parental mealtime strategies predict variations in diet quality

Since my suggestions are radical, driving the analysis toward a more linear clinical model, you should consult with the editor before revising the paper.

First, the Discussion places substantial interpretative weight on the multivariate regression model predicting diet quality (HEI-2020), despite the relatively modest proportion of explained variance. While a low-to-moderate R^2 is not uncommon in cross-sectional psychosocial research, particularly in heterogeneous neurodevelopmental populations, the current framing risks overstating the explanatory value of the model. + Diet quality in children with ASD is widely recognized as a multifactorial outcome shaped by neurodevelopmental severity, sensory sensitivities, gastrointestinal comorbidities, functional communication, adaptive skills, family routines, and access to interventions. Several recent reviews emphasize that feeding-related outcomes in ASD cannot be meaningfully interpreted without situating behavioral predictors within this broader clinical and contextual framework. In this light, the regression findings should be discussed more explicitly as exploratory and associative, rather than as evidence of robust predictive pathways.

We appreciate the reviewer’s insightful comment regarding the multifactorial nature of diet quality. We have revised the Discussion to explicitly state that factors such as neurodevelopmental severity, sensory sensitivities, and family routines are critical components of the nutritional landscape in ASD. In line with the reviewer's suggestion, we have reframed our regression findings as exploratory and associative, emphasizing that behavioral predictors cannot be interpreted in isolation from the broader clinical and contextual framework

Revised text (Discussion):

While the multivariate model identified BAMBI and PMAS as a significant predictor, it accounted for a modest 14.9% of the total variance. This suggests that diet quality in children with ASD is a complex, multifactorial outcome shaped by neurodevelopmental severity, sensory sensitivities, gastrointestinal comorbidities, and family routines. Consequently, our findings should be interpreted as exploratory associations rather than robust predictive pathways, as behavioral predictors must be situated within this broader clinical and contextual framework to be meaningfully interpreted.

Relatedly, the Discussion occasionally adopts language that implies directional or causal mechanisms (e.g., reinforcement cycles, parental strategies perpetuating poor diet quality). While such interpretations are theoretically plausible and well-grounded in behavioral and family-systems models, they are not directly testable within the present cross-sectional design. A more cautious framing emphasizing bi- directionality and transactional processes between child behavior and parental responses would better align the Discussion with both the study design and contemporary conceptual models highlighted in recent narrative and systematic reviews. + The interpretation of parental mealtime strategies warrants particular attention. The Discussion tends to characterize certain strategies as maladaptive, implicitly attributing a negative functional role to parental behaviors. However, this terminology risks pathologizing parental responses without sufficient empirical support, especially given the absence of

longitudinal or experimental data. Recent reviews on feeding interventions in ASD underscore that many parental accommodations emerge as adaptive short-term strategies in response to severe feeding refusal, sensory sensitivities, or medical concerns. Reframing these strategies as context-dependent responses rather than inherently maladaptive behaviors would add conceptual nuance and reduce the risk of causal oversimplification.

We have completely removed the term 'maladaptive' from the manuscript to avoid pathologizing parental responses. In line with the reviewer's suggestion, we have reframed parental accommodations as context-dependent, reactive strategies. We now emphasize that these behaviors often represent necessary short-term adaptive efforts to manage severe feeding challenges. The revised Discussion now explicitly situates these practices within a bi-directional and transactional framework, aligning with contemporary family-systems models and the limitations of our cross-sectional design

Revised text:

Consistent with our third hypothesis, parental mealtime strategies (PMAS) emerged as a significant independent predictor of children's diet quality. Interestingly, our multivariate regression model revealed that the total PMAS score had a significant positive effect on HEI-2020 scores ($\beta = 0.245$, $p = 0.015$). This suggests that structured and proactive parental interventions—such as ensuring daily fruit and vegetable availability or using positive persuasion—serve as protective factors that enhance overall diet quality. However, bivariate analyses highlighted the 'double-edged' nature of parental strategies: specific accommodative practices, such as frequently preparing 'special meals', were negatively correlated with diet quality ($r = -0.210$, $p = 0.028$). While preparing a child's preferred foods may function as a necessary short-term coping mechanism to ensure caloric intake in the face of severe food refusal, from a behavioral standpoint, this accommodation acts as an inadvertent reinforcement. Over time, this permissive loop perpetuates food selectivity, narrows the dietary repertoire, and ultimately drives the diet quality down. Therefore, interventions should empower parents to transition from reactive accommodations (e.g., special meals) to proactive, structured feeding routines.

The discussion of parental stress and quality of life is one of the strongest elements of the manuscript, yet it could be further strengthened by a clearer theoretical positioning. The findings suggest that parental stress is more closely linked to perceived burden and quality-of-life impairment than to isolated child feeding behaviors per se. This aligns with transdiagnostic and family-centered models described in recent reviews, which conceptualize caregiver stress as the product of cumulative demands rather than single child-level symptoms. Making this link more explicit would elevate the Discussion from a descriptive comparison to a theoretically informed interpretation. + Gender-related findings are discussed with interest, but the current narrative risks overinterpretation. Given the unbalanced sample size and age differences between boys and girls, the Discussion should consistently frame these results as exploratory and hypothesis-generating. Recent literature on gender differences in ASD emphasizes diagnostic and referral biases, as well as differential parental expectations, which may influence caregiver-reported stress. Integrating this perspective would provide a more balanced and literature-consistent interpretation.

We agree that the gender-related findings required a more cautious and literature-aligned interpretation. We have revised the discussion to explicitly frame these results as exploratory and hypothesis-generating, specifically citing the limitations of our unbalanced sample size and age differences. Furthermore, we have integrated the suggested perspectives on diagnostic biases and differential

parental expectations. This reframing ensures that our results are not overinterpreted as definitive sex differences but are instead situated within the broader context of caregiver perception and societal norms, as supported by recent ASD gender research

Revised text:

Our finding that parents of girls reported significantly higher stress and perceived burden (QoLA-B) compared to parents of boys must be interpreted with caution. Given the unbalanced gender distribution (85 boys vs. 26 girls) and the significant age differences between these groups, these results should be viewed as exploratory and hypothesis-generating rather than definitive. Rather than reflecting objective differences in symptom severity, these findings likely represent a complex interplay of sex-specific caregiver interpretations and societal norms. Future research with gender-matched cohorts is necessary to clarify whether these perceptions stem from distinct phenotypic profiles or varying psychosocial pressures associated with raising girls with ASD.

The use of HEI-2020 as a global diet quality indicator is appropriately highlighted as a strength, but the Discussion would benefit from a more explicit acknowledgment of its limitations in non-U.S. contexts. Recent reviews on nutritional assessment in ASD note that while composite indices are useful for comparative purposes, they may not fully capture culturally specific dietary patterns or clinically relevant nutrient inadequacies. Positioning HEI-2020 as a comparative, rather than normative, indicator within this sample would enhance methodological transparency and strengthen the credibility of the conclusions drawn.

We have revised the Discussion to acknowledge the methodological considerations of using the HEI-2020 in a Turkish context. We now explicitly position the index as a comparative tool rather than a normative standard, noting that composite indices may not capture all culturally specific dietary nuances.

Revised text (Discussion):

Our findings challenge these assumptions. It is important to note that the HEI-2020 is utilized here as a comparative rather than a normative indicator, as composite indices may not fully capture culturally specific dietary patterns or the clinical nuances of nutrient inadequacies in non-U.S. contexts. Rather than relying solely on descriptive parental reports of picky eating, our multivariate linear regression analysis provides robust quantitative evidence for this dietary deterioration. Even after adjusting for the child's age, BMI-z scores, and household income, the severity of mealtime behavioral problems emerged as a highly significant independent predictor of poorer diet quality. This demonstrates that the profoundly low HEI scores are directly driven by the core rigidities and sensory-based food refusals inherent to ASD 35,36, systematically narrowing the child's food repertoire to ultra-processed or nutrient-poor options.

Finally, the Discussion would be strengthened by a more explicit integration of recent systematic reviews published in the last five years on feeding disorders, ARFID, and diet quality in neurodevelopmental conditions. While several key studies are cited, the absence of a stronger review-based synthesis limits the interpretative depth of the section. Incorporating these reviews would not only contextualize the findings more robustly but also clarify how the present study advances the field beyond existing evidence.

We have significantly strengthened the Discussion section by integrating a robust synthesis of recent systematic reviews and meta-analyses published between 2023 and 2025. Specifically, we incorporated findings from Alhrbi et al. (2025)and Conti et al. (2025) to contextualize our diet quality results within the broader evidence of nutrient inadequacy and malnutrition risk in ASD.

Abstract: After revision, consider rewriting the abstract with statistical and design adjustments, specifying the role of HEI from 3-day food records.

We sincerely thank the reviewer for this constructive feedback. We have thoroughly revised the Abstract to accurately reflect the updated study design, methodology, and statistical findings. Specifically, as requested, we have explicitly clarified in the Methods section of the Abstract that the Healthy Eating Index-2020 (HEI-2020) scores were derived from 3-day food consumption records. Additionally, we have ensured that all statistical values reported in the abstract.

Introduction: Please include at least 5 recent reviews on ARFID/feeding disorder in neurodevelopmental conditions, multilevel interventions and behavioral on feeding problems in ASD, familial and stress outcomes associated with feeding problems. Also, the authors may include a review on diet quality in ASD to justify the utilization of HEI as the main outcome (diet quality in ASD, nutrient adequacy vs. patterns).

Following the reviewer's suggestion, we have updated the Introduction to include more than five recent systematic reviews (2023–2025) covering ARFID, sensory processing, and familial outcomes (e.g., Consoli et al., 2025; Archibald, 2023; Amate & Rosa, 2024; Chung et al., 2024; Conti et al., 2025). Furthermore, we have added a dedicated justification for utilizing the HEI as our primary outcome (In discussion). Citing recent meta-analytical evidence (e.g., Alhrbi et al., 2025), we argue that in ASD, assessing overall dietary patterns and quality is more clinically relevant than isolated nutrient adequacy, as it better captures the chronic selectivity and dietary rigidity characteristic of this population.

The manuscript furnishes data from a survey (not well specified in the text) in two clinical samples of ASD (preschool and primary). Data are collected by reliable measures, although self-reports. The introduction is lacking in literature. The hypotheses do not sufficiently guide the manuscript development. The survey data (table by sex) should be revised, explaining why the sample was split by sex (and why by sex only). Why were comorbidities not included in the model? Moreover, a deep revision of the data analysis section. Could the manuscript be reorganized moved several pieces of information, and the tables in the appendix or supplemental material? In my opinion, your proposal is publishable after major revisions, but you should take efforts to align hypothesis-research design-analysis. The main additional motive is the non-occidental sample and a more idiosyncratic mealtime context, but the availability of data could be more clinically guided.
